# Magnetic resonance angiography based on iron-coordinated poly(amino acid)s nanoprobes: an *in vitro* and *in vivo* experimental study

**DOI:** 10.1186/s41747-026-00829-5

**Published:** 2026-11-06

**Authors:** Tianlun Shen, Songyi Xu, Guangyao Li, Jingya Xia, Guocan Han, Yu Zhang, Yì Xiáng J. Wáng, Jun Ling, Jihong Sun

**Affiliations:** 1 Department of Radiology, Sir Run Run Shaw Hospital, School of Medicine, Zhejiang University, Hangzhou, China; 2 MOE Key Laboratory of Macromolecular Synthesis and Functionalisation, Department of Polymer Science and Engineering, Zhejiang University, Hangzhou, China; 3 Sir Run Run Shaw Hospital, School of Medicine, Zhejiang University, Hangzhou, China; 4 Department of Imaging and Interventional Radiology, Faculty of Medicine, The Chinese University of Hong Kong, Shatin, China

**Keywords:** Animals, Contrast media, Iron, Magnetic resonance angiography, Polysarcosine

## Abstract

**Objectives:**

To develop a gadolinium-free T1-weighted magnetic resonance (MR) contrast nanoprobe using Fe(III)-poly(amino acid)s-coordinated complexes (Fe@polyDOPA-*b*-polysarcosine, Fe@POS) for high-resolution MR angiography (MRA) with an extended imaging window.

**Materials and methods:**

Fe@POS nanoprobes were prepared by the self-assembly of Fe(III) and polyDOPA-*b*-polysarcosine copolymers. Physicochemical and cytological characterizations were conducted to demonstrate their feasibility for MRA. MRA of the aorta was conducted on New Zealand rabbits, which were divided into one experimental group (*n* = 3) receiving Fe@POS nanoprobes and one control group (*n* = 3) receiving Gadolinium diethylenetriamine pentaacetic acid (Gd-DTPA). Cardiac and hepatic MR acquisition were conducted on Bama Swine (*n* = 3). Long-term biodistribution (*n* = 3) and biosafety (*n* = 4) evaluation was performed on BALB/c mice. Short-term and acute toxicity evaluation was conducted on BALB/c mice (*n* = 5) and Bama pigs (*n* = 3).

**Results:**

Fe@POS nanoprobes exhibited a regular nanomicelle structure with an average particle size of 25 nm. Compared with Gd-DTPA (r_1_ = 4.38 mM^-1^ s^-1^), Fe@POS nanoprobes exhibited higher longitudinal relaxivity (r_1_ = 5.74 mM^-1^ s^-1^). In rabbit models, significant MR signal enhancement was observed in the abdominal aorta for over 4 h post-injection of Fe@POS. In porcine models, prolonged T1 enhancement was observed on MRA of the cardiovascular system, revealing detailed arterial and venous vasculature system in MR images. The biosafety evaluations demonstrated that the Fe@POS nanoprobes exhibited satisfactory biocompatibility and no prolonged iron accumulation in major organs under the tested conditions.

**Conclusion:**

Fe@POS nanoprobes simultaneously provided high-resolution T1-weighted MRA of the cardiovascular system with an extended imaging window.

**Key Points:**

***Question*** Can a gadolinium-free nanoprobe provide prolonged high-resolution T1-weighted magnetic resonance angiography?

***Findings*** The use of Fe (III)-based poly(amino acid)s nanoprobes enables high-spatial-resolution magnetic resonance angiography of the cardiovascular system with a long window period.

**Critical Relevance:**

The development of this new MRI contrast agent could broaden its clinical applicability and contribute to more precise diagnoses of cardiovascular diseases.

**Graphical Abstract:**

**Figure:**
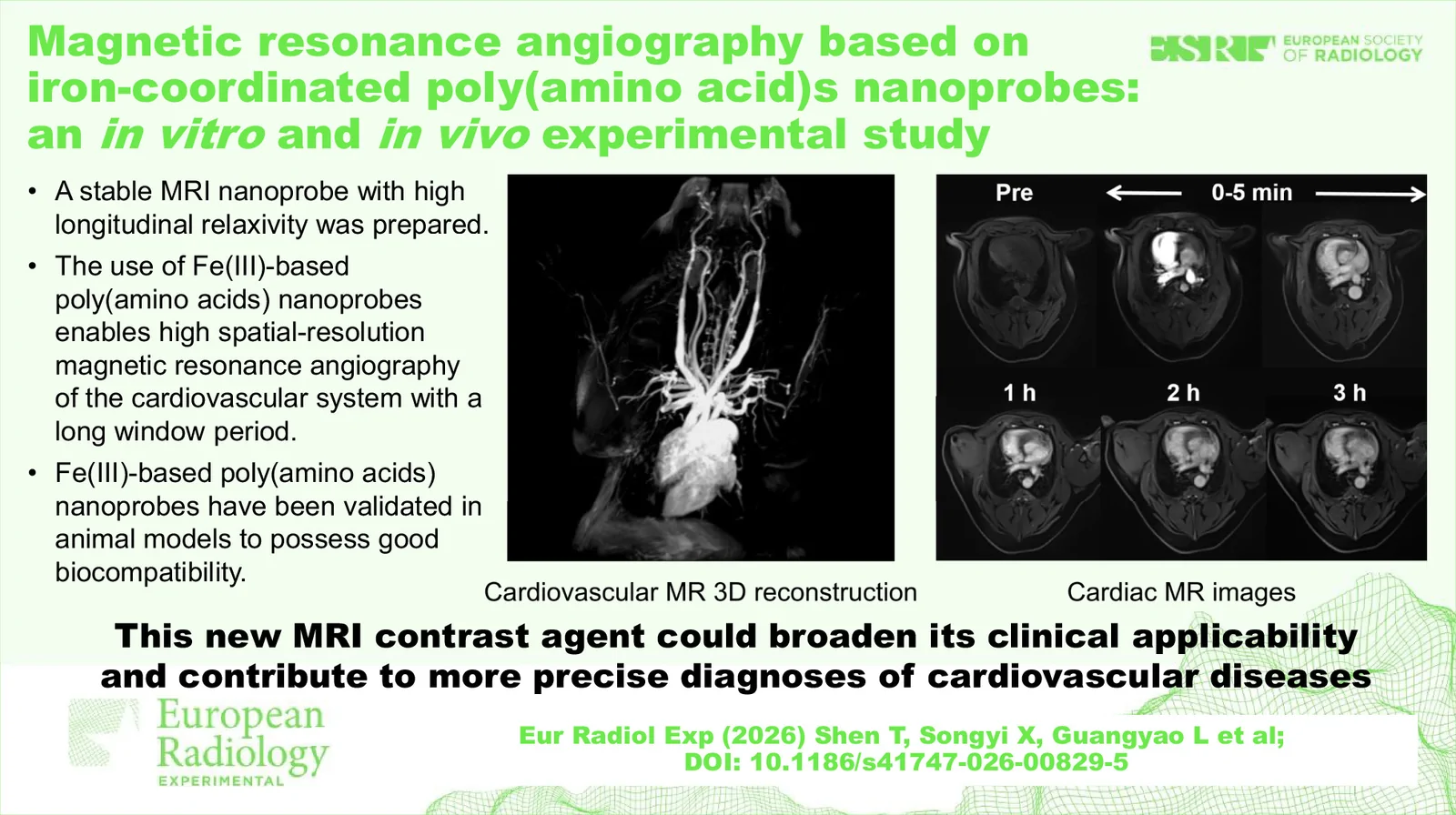

## Introduction

Cardiovascular diseases have emerged as major public health concerns, being the leading causes of death worldwide [1, 2]. Luminal and pathological changes typically occur during the pathogenesis of cardiovascular diseases and can be detected and differentiated using high-resolution angiography techniques [3]. In clinical practice, digital subtraction angiography (DSA) is considered the gold standard in the evaluation of vascular disorders, whereas DSA is an invasive intervention method with associated risk of complications [4, 5]. On the other hand, computed tomography angiography (CTA) provides detailed images of abnormal structures and physiological information regarding blood vessels [6]. However, the ionizing radiation generated by CTA raises concerns, especially for vulnerable populations such as pediatric and pregnant patients [7, 8].

Dynamic contrast-enhanced magnetic resonance angiography (MRA) has become an indispensable imaging technique for diagnosing cardiovascular diseases due to its high spatial resolution, unrestricted tissue penetration, non-invasive nature, and absence of ionizing radiation [9, 10]. Gadolinium-based contrast agents (GBCAs) dominate the medical applications of T1-weighted magnetic resonance (MR) contrast agents. Nevertheless, intravenously injected small molecular GBCAs rapidly extravasate from the blood capillaries into the tissue space, resulting in a short *in vivo* bloodstream circulation time and an insufficient imaging window [11, 12]. Additionally, GBCAs have been associated with potential nephrogenic systemic fibrosis in patients with renal dysfunction [13, 14] and deposition in brain tissues [15, 16]. These limitations warrant investigating more biocompatible and effective alternatives to GBCAs.

Researchers have developed Fe(III)-based MR contrast agents for multimodal MRA [17]. Fe(III), an essential trace element, has superior biosafety compared to Gd(III) [18]. Nevertheless, small molecular Fe(III) coordination complexes lacking inner-sphere water exchange sites display lower longitudinal relaxivity, similar to Gd(III) coordination complexes [17, 19–21]. Fe(III)-doped nanoparticles have longer tumbling time (τ_R_) and thus improve the longitudinal relaxivity compared to small molecular Fe(III) complexes. The significant enhancement is attributed to the geometrical confinement of Fe(III) in nanoparticles [22, 23]. Ferumoxytol, an ultra-small iron oxide formulation that is non-nephrotoxic, has been approved by the United States Food and Drug Administration for the treatment of iron-deficiency anemia. This formulation can noninvasively visualize acute myocardial infarction and aortic disease even in patients with advanced kidney disease [24]. Additionally, the bloodstream circulation time of Fe(III)-doped nanoparticles can be lengthened through appropriate hydrophilic surface modifications, contributing to long-term MR cardiovascular angiography [25–27].

Poly(α-amino acid)s are biocompatible and biodegradable polymers constructed using α-amino acid through peptide bond linkage [28]. The protein-mimicking structures and properties of poly(α-amino acid)s have driven the widespread applications in biomedical fields, including stimuli-responsive biomaterials [29], controlled drug and gene delivery [30, 31], tissue engineering [32], and biomedical imaging [33, 34]. Polysarcosine, a member of the polypeptide family, possesses high water solubility, low cellular toxicity, protein resistance, and no immunogenicity [35].

In this study, we aimed to develop a gadolinium-free T1-weighted contrast agent for high-resolution and long-term MRA of the cardiovascular system. We report the preparation of a Fe(III)-polymer-coordinated nanoprobe (Fe@POS) by self-assembling Fe(III) with a polyDOPA-*b*-polysarcosine diblock copolymer, designed to enable accurate MRA with a long time window. Furthermore, we evaluated the *in vivo* biocompatibility of Fe@POS nanoprobe to elucidate its potential clinical application.

## Materials and methods

### Study design

This study was conducted under the supervision and guidelines of the Ethics Committee of the Affiliated Sir Run Run Shaw Hospital, School of Medicine, Zhejiang University. MRI experiments were performed on 3-T MR scanners. Animal experiments followed the protocols and standards for the care and use of experimental animals according to the local animal welfare committee (Hangzhou Province, China; approval number: SRRSH202310001). All animal experiments were conducted under the guidance and supervision of a certified laboratory animal veterinarian (Y.Y.). BALB/c mice (*n* = 3 for long-term biodistribution and *n* = 4 for biosafety evaluation, Zhejiang Vital River Laboratory Animal Technology Co. Ltd.), New Zealand Rabbits (*n* = 6; Fe@POS group, *n* = 3; Gd-DTPA control group, *n* = 3. Zhejiang Vital River Laboratory Animal Technology Co. Ltd.) and Bama miniature pigs (*n* = 3, Zhejiang Vital River Laboratory Animal Technology Co. Ltd.) were included in this study. An overview of all *in vitro* and *in vivo* experiments conducted in this study is provided in Table 1.

**Table 1:** Overview of *in vitro* and *in vivo* experiments included in this study

	Experiment	Instrument	Model
*In vitro*	Nanoparticle morphology analysis	Electron transmission microscope (JEM-1400plus, Japan Electron Optics Laboratory)	Fe@POS nanoparticles
	Particle size measurement	Dynamic laser scattering analyzer (Zetasizer Nano Series, Malvern Instruments)	Fe@POS nanoparticles
	Relaxivity measurement	3.0-T MR scanner (SignaHDxt, GE Medical Systems)	Fe@POS nanoparticles
	Cellular uptake assay	Flow cytometer (CytoFLEX LX, Beckman)	CT26 cells
	Cytotoxicity assay	Microplate reader (BIO-RAD)	NIH 3T3 cells
*In vivo*	Rabbit magnetic resonance angiography (MRA)	3.0-T MR scanner (SignaHDxt, GE Medical Systems)	New Zealand rabbits (*n* = 6)
	Porcine MRA	3.0-T MR scanner (Siemens MAGNETOM Vida, Siemens Medical Solutions)	Bama swine (*n* = 3)
	Biodistribution analysis	Inductively coupled plasma-mass spectrometry (Thermo XSeries II, Thermo Fisher Scientific)	BALB/c mice (*n* = 3 per group)
	Long-term biosafety analysis	Body weight monitoring	BALB/c mice (*n* = 4 per group)
	Serum biochemical & hematological analysis	Automatic biochemical analyzer (AU480, Beckman)	BALB/c mice (*n* = 4 per group)
	Short-term/acute toxicity	Automatic biochemical analyzer (AU480, Beckman)	BALB/c mice (*n* = 5 per group) & Bama swine (*n* = 3 per group)

### Synthesis and characterization of Fe@POS nanoprobe

Fe@POS nanoprobes were synthesized by self-assembly of Fe(III) with polyDOPA-*b*-polysarcosine diblock copolymer (PDOPA-*b*-PSar, abbreviated as POS). Detailed synthesis protocol, including monomer synthesis, copolymerization, and self-assembly process, is provided in Supplementary Material 1.

The Fe(III) concentrations were measured by inductively coupled plasma-mass spectrometry (ICP-MS) on a Thermo XSeries II spectrometer (Thermo Fisher Scientific, Waltham, USA). The dynamic light scattering (DLS) measurements were carried out using a particle size analyzer (Zetasizer Nano Series, Malvern Instruments). Transmission electron microscope (TEM) measurements were carried out using a JEM-1400plus electron microscope (Japan Electron Optics Laboratory).

### Cell culture

The mouse-derived colon carcinoma (CT26) cells and mouse embryonic fibroblast (NIH 3T3) cells (American Tissue Type Culture Collection, Manassas, USA) were cultured in high-glucose Dulbecco’s modified Eagle’s medium (DMEM) containing 4.0 mM L-glutamine (Gibco), supplemented with 10% fetal bovine serum and 1% penicillin/streptomycin. All cells were cultivated at 37 °C in 5% CO_2_ atmosphere using a cell humidified incubator.

### *In vitro* cytotoxicity and cellular uptake assay

Cytotoxicity was evaluated in NIH 3T3 cells using the Cell Counting Kit-8 assay after 24 h exposure to POS and Fe@POS at concentrations ranging from 10 to 1,000 µg/mL, as described in Supplementary Material 2. Cellular uptake of Fe@POS was assessed in CT26 cells using FITC-labeled Fe@POS *via* flow cytometry and confocal microscopy, as described in Supplementary Material 3.

### MRI analysis

Magnetic resonance scanning was performed on a 3-T MR scanner (SignaHDxt, GE Medical Systems). Longitudinal relaxivity r_1_ was obtained by an inversion recovery pulse sequence with variable inversion times. All measurements of longitudinal relaxivity r_1_ were performed at a temperature of 20 °C and a magnetic field strength of 3.0 T. Detailed protocols for relaxivity measurements are provided in Supplementary Material 4.

*In vivo* MRI of New Zealand rabbits was performed using a 3-T clinical human MR system (SignaHDxt, GE Medical Systems) and abdominal coil to simulate the scanning condition of patients. A total of six New Zealand rabbits (3.17 ± 0.35 kg body weight) were divided into two independent groups (*n* = 3 per group). One group received Fe@POS (0.1 mmol Fe/kg) as the experimental group, and the other group received Gd-DTPA (0.1 mmol Gd/kg) as the control group. The MR images were acquired before and after administration at different time points. The MR signal of the aorta in New Zealand rabbits was measured. Contrast-to-noise ratio (CNR) values and aorta signal retention values were calculated, as described in Supplementary Material 5.

*In vivo* MRA of Bama swine models was performed on a 3-T scanner (Siemens MAGNETOM Vida, Siemens Medical Solutions). Bama swine models (*n* = 3, 30.3 ± 2.1 kg body weight) were intravenously administered with Fe@POS nanoprobes (0.06 mmol Fe/kg). The MR signals of the thoracic aorta and hepatic vein were acquired before and after Fe@POS administration at 0, 1, 2, and 3 h. CNR values of the thoracic aorta and hepatic vein were calculated, as described in Supplementary Material 6.

### Biosafety study

For the biodistribution analysis, female BALB/c mice (22.4 ± 1.3 g body weight, mean ± standard deviation) were randomly divided into five experimental groups (*n* = 3 for each experimental group) and one control group (*n* = 3). The mice of the experimental group were intravenously administered with Fe@POS (0.1 mmol Fe/kg), whereas the mice of the control group were intravenously injected with an equal volume of saline. The Fe concentration of major organs (heart, lung, liver, spleen, kidney) was quantified by inductively coupled plasma-mass spectrometry (ICP-MS), as described in Supplementary Material 7.

To evaluate the biosafety of Fe@POS, long-term body weight monitoring was conducted. Healthy female BALB/c mice (21.1 ± 1.0 g body weight) were randomly divided into three groups: a control group (*n* = 4) receiving saline, a standard-dose group (*n* = 4) receiving Fe@POS (0.1 mmol Fe/kg), and a high-dose group (*n* = 4) receiving Fe@POS (0.3 mmol Fe/kg). All injections were administered intravenously every 3 days. The body weight of the mice was recorded every day. Serum biochemical and hematological analysis were performed to investigate the chronic and acute toxicity of Fe@POS. Detailed experimental procedures are provided in Supplementary Material 8.

### Statistical analysis

Data were presented as mean ± standard deviation (SD) unless otherwise stated and analyzed by GraphPad Prism V8.0. The CNR values and signal retention rates at different time points of the Fe@POS experimental group and control group were compared using two-way mixed analysis of variance (ANOVA) followed by Šídák’s multiple comparisons test. The body weight of three mice groups at day 14 was compared using one-way ANOVA with Dunnett’s correction. The biochemical and hematological indexes of three mice group was compared using one-way ANOVA with Dunnett’s correction. Comparisons of CNR values at different time points within the same group were performed using one-way repeated-measures ANOVA with Geisser-Greenhouse correction when Mauchly’s test of sphericity indicated violation of the sphericity assumption. A *p*-value < 0.05 was considered statistically significant (*p < 0.05, **p < 0.01, ***p < 0.001).

## Results

### Synthesis and characterization of Fe@POS

**Fig. 1:**
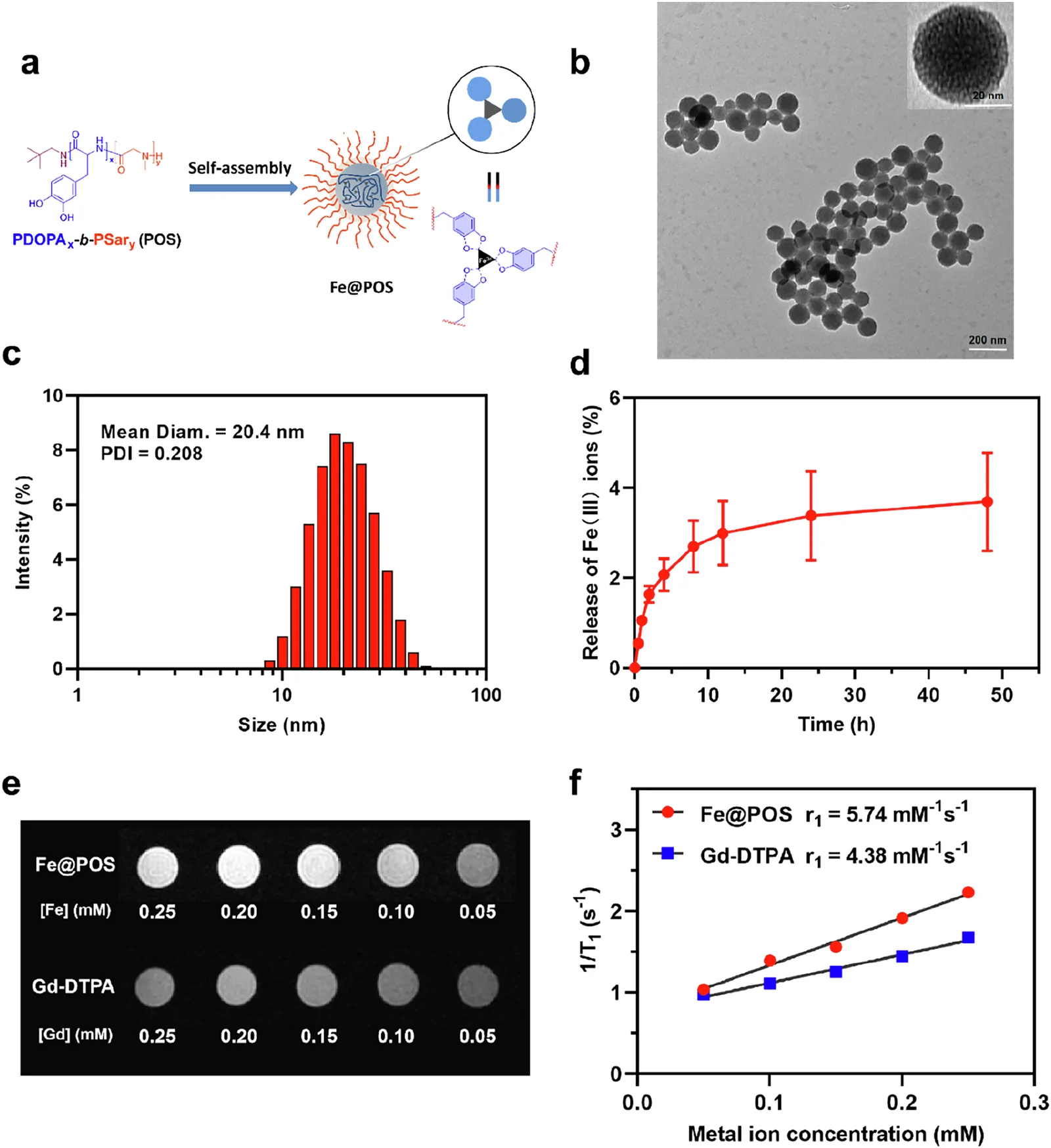
Physiochemical characterization of Fe@POS. **a** Schematic illustration of the self-assembly of Fe@POS nanoprobes. **b** Transmission electron microscope images of Fe@POS nanoparticles. Scale bar: 200 nm. **c** The size distribution of Fe@POS nanoparticles. **d** The release profile of Fe(III) ions within 48 h after synthesis of Fe@POS (*n* = 3). **e** T1-weighted images of Fe@POS and Gd-DTPA at different Fe/Gd concentrations. **f** Linear fitting of the reciprocal of T1 relaxation time to metal ion concentration at the magnetic field of 3.0 T

PolyDOPA-*b*-polysarcosine (PDOPA-*b*-PSar, abbreviated as POS) diblock copolymer was synthesized *via* ring-opening polymerization of *N*-phenyloxycarbonyl-3,4-dihydroxy-L-phenylalanine (L-DOPA-NPC) and *N*-phenyloxycarbonyl-sarcosine (Sar-NPC) initiated by neopentylamine (Supplementary Figs. 1–5). Size exclusion chromatography analysis showed a decrease in retention time from PDOPA to POS, indicating complete initiation of Sar-NPC polymerization by the terminal amine groups of PDOPA (Supplementary Fig. 6). POS can spontaneously self-assemble into amphiphilic polymeric micelles in the aqueous phase. Moreover, it can chelate Fe(III) *via* the catechol groups on the polyDOPA side chains (Fig. 1a). The Fe encapsulation efficiency and loading capacity were 27.94 ± 4.14% and 1.20 ± 0.17%, respectively, determined *via* inductively coupled plasma-optical emission spectrometry.

The physicochemical properties of Fe@POS are summarized in Table 2. The Fe@POS nanoprobes were spherical with a uniform diameter of approximately 25 nm, and a sophisticated, facile micelle structure was observed (Fig. 1b). Dynamic light scattering analysis suggested that the mean hydrodynamic size of Fe@POS was 20.4 nm, with a narrow polydispersity index (PDI) of 0.208 (Fig. 1c), consistent with the transmission electron microscopy results. Additionally, the Fe@POS nanoprobes exhibited long-term stability in phosphate buffer solution (PBS) and saline containing endogenous cations (Zn(II), Ca(II), Mg(II)), without substantial changes in hydrodynamic size and longitudinal relaxivity within 10 days after fabrication (Supplementary Fig. 7a, b). To verify the stability of Fe@POS under physiological conditions, serum extracted from BALB/c mice was co-incubated with Fe@POS at 37 °C for 48 h. The results showed no significant changes in the particle size and relaxivity of the Fe@POS nanoprobes (Supplementary Fig. 7c, d). The release profile of Fe@POS demonstrated that the cumulative release of Fe(III) ions from the nanoparticle interior was below 5% within 48 h post-synthesis (Fig. 1d). The above results indicate highly stable chelation between the Fe(III) ions and the catechol groups in the core of Fe@POS nanoprobes.

**Table 2:** Physicochemical properties of Fe@POS nanoprobe

Parameter	Fe@POS
Fe encapsulation efficiency	27.94 ± 4.14%
Fe loading capacity	1.20 ± 0.17%
Morphology (characterized by TEM)	Spherical particles with uniform size
Particle size (measured by TEM)	25 nm
Hydrodynamic size (measured by DLS)	20.4 nm
Polydispersity index	0.208
Fe(III) release	< 5%
Longitudinal relaxivity (mM^-1^ s^-1^)	5.74

*DLS* Dynamic light scattering, *TEM* Transmission electron microscope

*In vitro* T1-weighted MRI suggested that the T1 signal intensity increased with the Fe concentration (Fig. 1e). The longitudinal relaxivity r_1_ of Fe@POS and Gd-DTPA was measured at a temperature of 20 °C, a magnetic field strength of 3.0 T, and in deionized water. The results showed that the r_1_ value of Fe@POS was 5.74 mM^-1^·s^-1^ (Fig. 1f), which is 1.3-fold higher than that of Gd-DTPA (4.38 mM^-1^·s^-1^).

### Cytotoxicity and intracellular uptake of Fe@POS

**Fig. 2:**
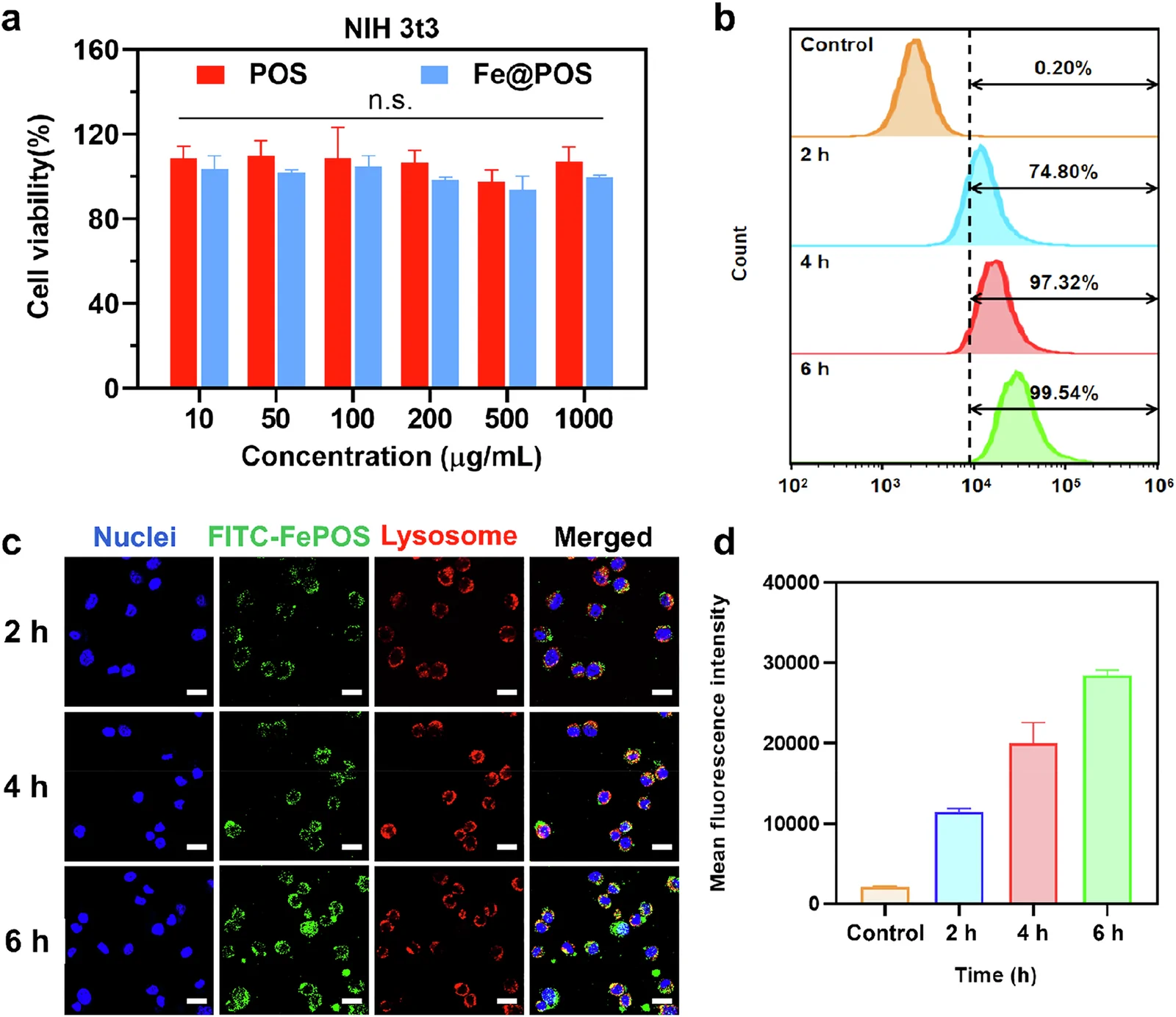
Cell viability of POS and Fe@POS and subcellular distribution and cellular uptake of Fe@POS. **a** Cell viability of NIH 3T3 cells treated with POS and Fe@POS (*n* = 3 biologically independent samples). **b** Flow cytometry assay of Fe@POS labeled with FITC showing the cellular uptake of CT26 cells after different incubation times. **c** Confocal laser scanning microscope images of CT26 cells treated with Fe@POS for different incubation times. Scale bar: 20 μm. **d** Mean fluorescence intensity of flow cytometry assay of Fe@POS in **b**

The intracellular behavior of Fe@POS, focusing on biocompatibility and cellular uptake, was investigated before MRI analysis. The cytotoxicity of POS and Fe@POS was evaluated. No statistically significant cytotoxicity was observed for either POS or Fe@POS across the tested concentrations from 10 μg/mL to 1,000 μg/mL after 24 h of incubation (Fig. 2a).

Flow cytometry was conducted to analyze the cellular uptake of fluorescein isothiocyanate (FITC)-labeled Fe@POS. The extension of incubation time from 2 h to 6 h increased the cellular uptake ratio of CT26 cells from 74.80% to 99.54%, indicating that CT26 cells rapidly absorbed FITC-Fe@POS within a relatively short time (Fig. 2b, d). Furthermore, confocal microscopy fluorescence imaging suggested the subcellular distribution of FITC-Fe@POS (Fig. 2c). Green FITC fluorescence was observed in the cytoplasm, overlapped with red fluorescence of the lysosomes. The co-localization of FITC-Fe@POS and LysoTracker in the merged pictures suggested that the cellular uptake of Fe@POS nanoprobes was primarily mediated by the endolysosomal pathway (Supplementary Fig. 8).

### *In vivo* T1-weighted MRI with Fe@POS in rabbit and porcine models

**Fig. 3:**
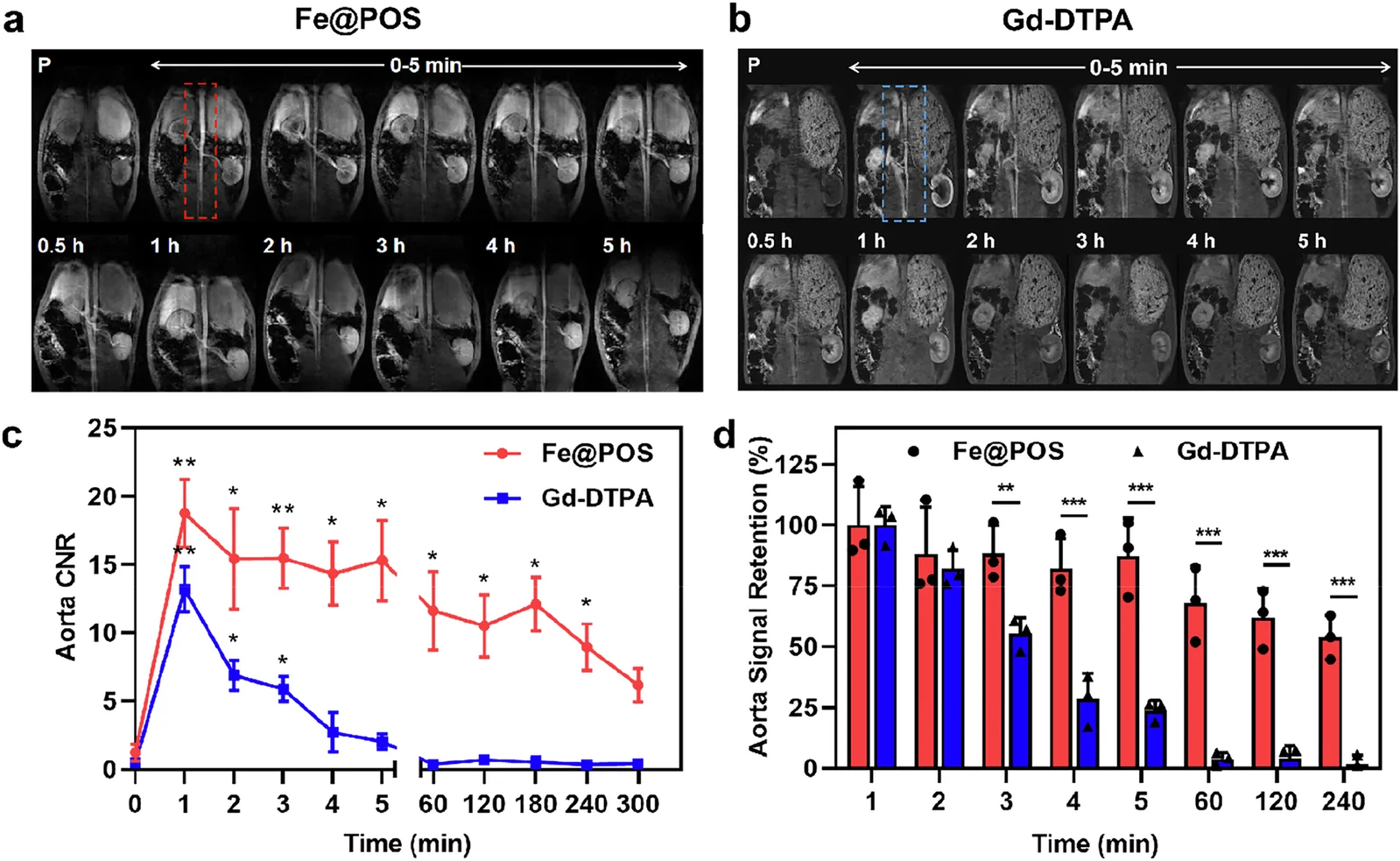
*In vivo* MR images of rabbit models at different times after treatment with Fe@POS (experimental group, *n* = 3) or Gd-DTPA (control group, *n* = 3). Representative coronal T1-weighted MR images obtained at different times after Fe@POS nanoprobes administration (**a**) or Gd-DTPA administration (**b**). The aortas are identified by a red dashed box (Fe@POS) and a blue dashed box (Gd-DTPA). **c** CNR curve of the rabbit aorta over time. **d** Aorta signal retention curve of the rabbit aorta over time. CNR, Contrast-to-noise ratio

In the blood pool MRA of the rabbit model, comparative analysis of contrast enhancement kinetics was conducted between Fe@POS and Gd-DTPA as a control (Fig. 3a, b). The ROIs used to evaluate the T1 signal intensity of the aorta and kidneys are shown in Supplementary Fig. 9. Quantitative evaluation revealed superior performance of Fe@POS compared to Gd-DTPA in both aortic enhancement magnitude and duration. The Fe@POS group showed a significantly higher aortic contrast-to-noise ratio (CNR) when compared to the Gd-DTPA group: aortic CNR value was 18.8 ± 2.5 for the Fe@POS group and 13.1 ± 1.6 for the Gd-DTPA group (*p* = 0.039) 1-min after injection (Fig. 3c). The temporal evolution of contrast enhancement demonstrated differences between the two MRI contrast agents. Aortic CNR value of the Gd-DTPA group returned to baseline levels within 4 min post-injection, while the Fe@POS group maintained significantly elevated aortic CNR values during 4 h post-administration. As demonstrated in Fig. 3d, the Fe@POS group exhibited markedly slower signal decay kinetics relative to the Gd-DTPA group. Quantitative analysis demonstrated higher aortic signal retention in Fe@POS group from 3-min time point (Fe@POS 88.1 ± 11.6% *versus* Gd-DTPA 43.5 ± 6.6%, *p* = 0.008) to 240-min time point (Fe@POS 53.7 ± 9.0% *versus* Gd-DTPA 1.6 ± 3.7%, *p* < 0.001) (Fig. 3d). Additionally, the CNR of rabbit kidney of Fe@POS group increased from 0.54 ± 0.04 to 4.22 ± 0.47 (*p* = 0.005) within 5 min post-injection and remained elevated for over 5 h. Comparatively, in the Gd-DTPA control group, the kidney CNR peaked at 1-min post-injection (0.27 ± 0.05 to 2.47 ± 0.80, *p* = 0.031), then progressively decreased and returned to baseline within 3 h (Supplementary Fig. 10).

**Fig. 4:**
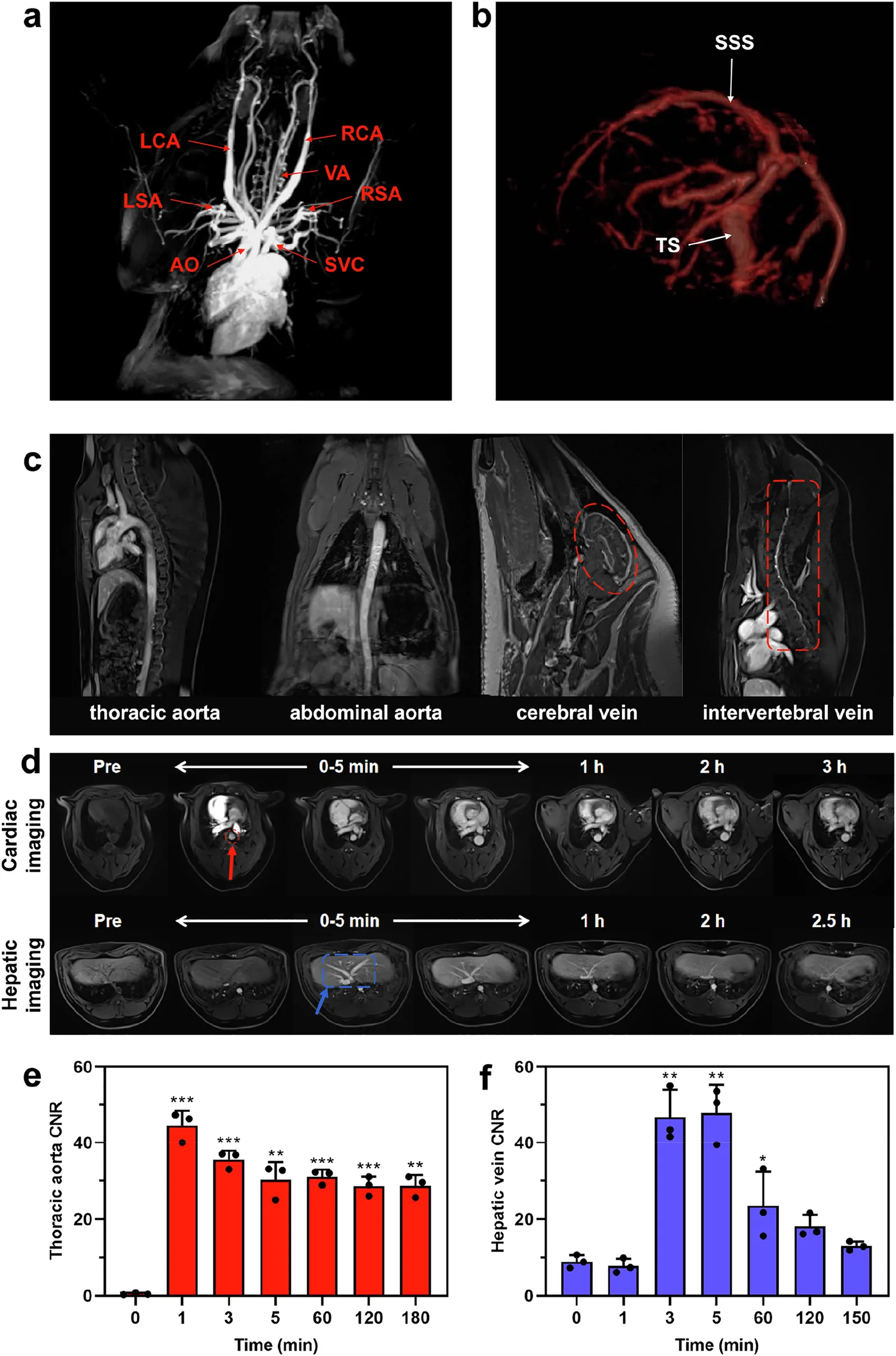
*In vivo* MR study of Fe@POS using Bama porcine models (*n* = 3). **a** Cardiovascular three-dimensional reconstruction of MRA. **b** Cerebrovascular three-dimensional reconstruction of cerebral venous sinuses. **c** Images of the thoracic aorta, abdominal aorta, cerebral vein (red dashed circle), and intervertebral vein (red dashed box). **d** Cardiac and hepatic images at different times. Thoracic aortas are identified by the red arrow and dashed circle. Hepatic veins are identified by the blue arrow and dashed box. CNR changes of the thoracic aorta (**e**) and hepatic vein (**f**) over time. AO, Aorta; CNR, Contrast-to-noise ratio; L/RCA, Left/right carotid artery; L/RSA, Left/right subclavian artery; VA, Vertebral artery; SVC, Superior vena cava; SSS, Superior sagittal sinus; TS, Transverse sinus

MRA was also performed in the Bama swine model (*n* = 3). After intravenous injection, the Fe@POS nanoprobes circulated to the heart through the bloodstream. They were pumped out through the aorta to synchronously enhance the heart, arteries, and veins. The upper body of a swine displays a high spatial resolution of the vasculature using Fe@POS-enhanced MRA, which facilitated identifying sophisticated blood vessels of different levels (Fig. 4a, c). The primary arteries of the heart include the aorta, carotid artery and its branches, subclavian artery and its branches, and vertebral artery. The major veins returning to the heart include the superior vena cava and inferior pulmonary vein. The cerebral venous sinuses, including the transverse sinus, superior sagittal sinus, inferior sagittal sinus, and superior cerebral veins, were visualized within 2 h post-injection of the Fe@POS nanoprobes (Fig. 4b). Additionally, the Fe@POS nanoprobes enabled prolonged cardiac and hepatic T1-weighted MRI. Cardiac MRI analysis revealed significant enhancement of thoracic aorta signal after Fe@POS administration, with sustained contrast maintenance exceeding 3 h (Fig. 4d). The ROIs used to evaluate the T1 signal intensity of thoracic aorta and hepatic vein are shown in Supplementary Fig. 11. Quantitative assessment demonstrated a significant elevation in aortic CNR from 0.5 ± 0.2 to the peak value of 44.5 ± 3.9 within 1 min (*p* < 0.001). The aortic CNR remained significantly elevated at 28.7 ± 2.8 after 3 h compared to pre-injection CNR (*p* < 0.001, Fig. 4e). Hepatic venous enhancement initiated at 3 min post-injection and persisted for more than 60 min. The hepatic venous CNR increased significantly from 8.9 ± 1.6 to the peak value of 47.8 ± 7.4 (*p* = 0.003), with a gradual decline to 23.5 ± 8.9 at 1 h compared to pre-injection CNR (*p* = 0.047) (Fig. 4f).

### Biosafety and biodistribution of Fe@POS

**Fig. 5:**
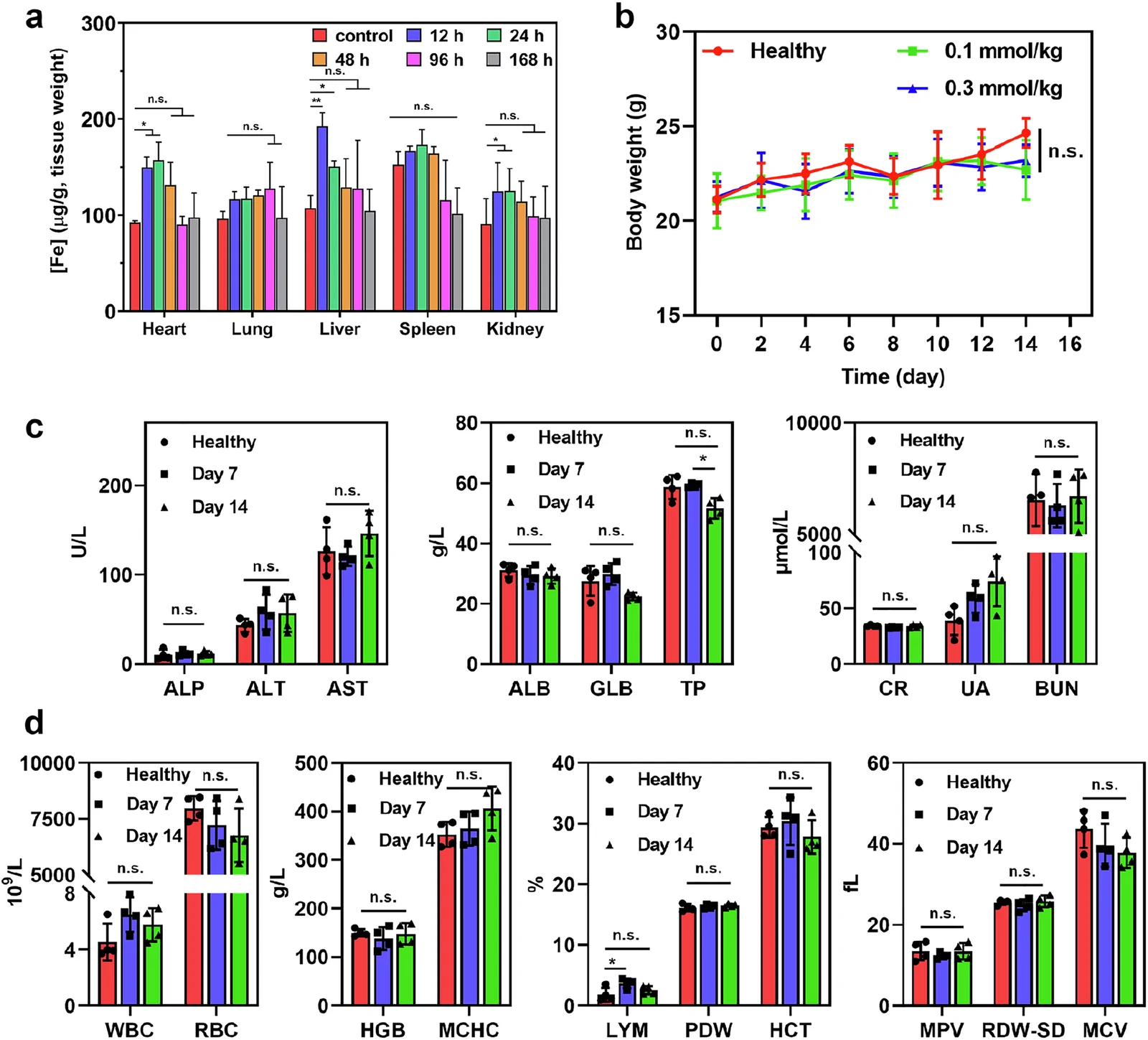
Long-term systemic biodistribution and biotoxicity evaluation of Fe@POS in female BALB/c mice. **a** Quantitative biodistribution of Fe@POS in mice (*n* = 3) after intravenous injection (0.1 mmol Fe/kg) at 12, 24, 48, 96, 168 h. **b** Body weight curves in mice (*n* = 4) receiving saline (healthy), standard dose (0.1 mmol Fe/kg), or high dose (0.3 mmol Fe/kg) of Fe@POS every 3 days. **c** Serum biochemical analysis of hepatotoxicity (parameters: ALT, AST, ALP, ALB, GLB, TP) and nephrotoxicity (parameters: CR, UA, BUN) of healthy mice (*n* = 4) and Fe@POS-treated mice (*n* = 4) at day 7 and day 14. Mice were intravenously injected with saline (healthy) or Fe@POS (0.3 mmol Fe/kg) every 3 days. **d** Hematological analysis of peripheral blood in mice (*n* = 4) treated with Fe@POS (0.3 mmol Fe/kg) every 3 days. ALB, Albumin; ALP, Alkaline phosphatase; ALT, Alanine aminotransferase; AST, Aspartate aminotransferase; BUN, Blood urea nitrogen; CR, Creatinine; GLB, Globulin; HCT, Hematocrit; HGB, Hemoglobin; LYM, Lymphocyte count; MCHC, Mean corpuscular hemoglobin; MCV, Mean corpuscular volume; MPV, Mean platelet volume; PDW, Platelet distribution width; RBC, Red blood cells; RDW-SD, Red blood cell distribution width; TP, Total protein; UA, Uric acid; WBC, White blood cells

To systematically evaluate the *in vivo* behavior of Fe@POS nanoprobes, we conducted biodistribution analysis in BALB/c mice. Quantitative ICP-MS revealed preferential accumulation of Fe@POS in the heart, liver, and kidney tissues within 24 h post-injection (Fig. 5a). The iron concentration in all examined organs (heart, lung, liver, spleen, and kidney) showed no significant difference compared to the control group by 48 h, demonstrating that Fe@POS does not cause prolonged iron accumulation in major organs.

The biotoxicity of Fe@POS has also been investigated. We additionally established a high-dose group (three times the standard dose) to investigate whether excessive administration of Fe@POS would induce systemic toxicity. Both standard and high-dose groups presented negligible fluctuations in average body weights throughout the 14-day observation period (Fig. 5b), with no statistically significant difference in comparison with healthy mice, indicating the absence of systemic toxicity. According to serum biochemical analysis (Fig. 5c), all measured liver function parameters (alanine aminotransferase ALT, aspartate aminotransferase AST, alkaline phosphatase ALP, albumin ALB, globulin GLB, total protein TP) and kidney function parameters (creatinine CR, uric acid UA, blood urea nitrogen BUN) remained within physiological reference ranges after 7 or 14 days, demonstrating the absence of Fe@POS-induced hepatic and renal toxicity. The biosafety of Fe@POS was further validated *via* hematological analysis in both rodent and porcine models. In the long-term repeated-dose study in mice, no significant differences in hematological parameters were observed at day 7 or day 14 compared to the control group (Fig. 5d). In the short-term and acute toxicity study, no significant changes in hematological parameters were detected in mice at 24 h post-injection, nor in pigs at 24 h and 48 h post-injection of Fe@POS (Supplementary Figs. 12 and 13). All the cumulative evidence demonstrated the excellent biosafety of Fe@POS nanoprobes.

## Discussion

The short *in vivo* bloodstream circulation time (< 30 min) of GBCAs represents a clinical limitation when applied to MRA, as it restricts the temporal window for vascular imaging. Additional concerns include the risk of nephrogenic systemic fibrosis in patients with renal dysfunction and long‑term tissue deposition of gadolinium [11–14]. This necessitates the development of novel magnetic resonance contrast agents with high biosafety and long vascular retention time. In this study, we designed and fabricated self-assembled nanomicelles (Fe^3+^@PDOPA-*b*-PSar, Fe@POS) based on an amphiphilic polyDOPA-*b*-polysarcosine diblock copolymer demonstrating high longitudinal relaxivity (r_1_ = 5.74 mM^-1^ s^-1^, 20 °C, 3.0-T magnetic field and in deionized water). It should be noted that the r_1_ value may differ under physiological conditions *in vivo*. In animal models, the Fe@POS nanoprobes remain in the vasculature for more than 3 h and enable high-spatial-resolution MRA.

The characteristics of dynamically enhanced MR after Gd-DTPA administration are often described as ‘fast forward and fast out’ [36]. In clinical practice, contrast-enhanced MRA requires precise bolus timing to synchronize the acquisition with peak arterial enhancement. Early imaging, when the contrast agent first arrives, can lead to ringing artifacts and decreased signal intensity. Conversely, late imaging, toward the tail end of the contrast agent, results in reduced signal intensity due to venous and soft tissue enhancement [37]. However, imaging with a long time window is essential in clinical practice because several cardio-cerebrovascular diseases involve multiple organs throughout the body, such as atherosclerosis and arterial aneurysms [38, 39]. In this study, the MRI results suggested that the retention time of Fe@POS nanoprobes in the bloodstream was 4 h in rabbit models and > 3 h in porcine models, surpassing that of Gd-DTPA. Additionally, it is challenging to distinguish a sophisticated vascular system, particularly for microvessels not aligned perpendicularly to the scanning orientation, in dynamically enhanced MRA. In our study, 3D MRA reconstruction of the porcine models after Fe@POS administration revealed distinct arterial and venous system morphology. This improvement was attributed to the prolonged vascular retention and high MR sensitivity of the Fe@POS nanoprobes. Therefore, the Fe@POS nanoprobes offer superior diagnostic precision compared to clinical GBCAs, as they can simultaneously and accurately image both arteries and veins.

With the development of nanotechnology, clinicians have utilized biomedical molecular nanoprobes to accurately image blood vessels and visualize pathological processes within lesions and surrounding tissues [40]. In addition to the GBCAs, ferumoxytol, a superparamagnetic iron oxide (SPIO)-based formulation, has been reported as a T1-weighted contrast agent in MRA [41, 42]. Both ferumoxytol and Fe@POS are Fe(III)-based nanoparticles, but their *in vivo* distributions and imaging patterns are notably different. Ferumoxytol exhibits dual T1 and T2 effects, significantly attenuating the T1 signal at high concentrations. Therefore, susceptibility artifacts mimicking vascular thrombosis can be observed in the abdominal aorta during the first-pass window [43]. Different from ferumoxytol, Fe@POS nanoprobe derives its MRI enhancement capabilities exclusively from the paramagnetic Fe(III) ions densely clustered in the core of nanomicelles. The unique nano-structure of Fe@POS, lacking crystalline lattice and superparamagnetism that cause T2 MR enhancement, enables robust T1 MR enhancement effect at relatively high concentrations, which is an advantage compared to ferumoxytol. Additionally, one of the unresolved clinical challenges faced by these nanosized SPIO nanoparticles is their potential to induce hypersensitivity reactions. Studies have shown that SPIO-based nanoparticles can activate the complement system upon entering the human body, thereby triggering complement activation-related pseudoallergy [44, 45]. These hypersensitivity reactions, including dyspnea, fever, rash, and palpitations, have contributed to the withdrawal of the majority of commercial SPIO-based contrast agents from the market (ferumoxide/Endorem^®^, ferumoxytol/Feraheme^®^) [46]. Fe@POS is chemically distinct from SPIO-based nanoparticles. Fe@POS employs a polysarcosine shell—a highly hydrophilic polypeptide known to resist non‑specific protein adsorption and suppress complement activation [35, 47]. This coating contrasts sharply with the carbohydrate or synthetic polymer coatings used in earlier SPIO-based formulations, which have been implicated in complement activation-related pseudoallergy. In line with this design, our biosafety evaluation in mice and swine revealed no acute adverse reactions. Thus, we hypothesize that Fe@POS may not elicit the same hypersensitivity reaction as SPIO-based contrast agents, because the polysarcosine shell of Fe@POS prevents complement activation.

In clinical MRI applications, conventional low-molecular-weight GBCAs are constrained by the rapid systemic clearance, which necessitates the use of fast scanning sequences to capture images during the first pass of GBCAs through the vessels. In this study, the prolonged bloodstream retention of Fe@POS enables the implementation of extended scanning sequences, offering substantial advantages for imaging complex and small vessels. The current study is in the preclinical trial phase. To facilitate the clinical translation of Fe@POS as an MRA contrast agent, future work will focus on comprehensive data collection including multi-model evaluation of long-term *in vivo* imaging performance.

Our study has several limitations. First, while we have demonstrated the feasibility and imaging efficacy of Fe@POS nanoprobes in animal models, the optimal dosage was not investigated. The appropriate dose for MRA may vary considerably depending on body weight, metabolic rate, and hemodynamic characteristics of each animal species. Establishing the relationship between dosage and imaging efficacy in large animal models is essential for the clinical translation of the Fe@POS nanoprobe. Second, the potential phagocytosis of Fe@POS nanoprobes by vascular endothelial cells was not evaluated in the present study. Endothelial internalization of circulating nanoparticles could significantly influence their intravascular residence time and imaging kinetics. Given that prolonged vascular retention is a key advantage of Fe@POS nanoprobe for MRA, understanding the interaction between the nanoprobe and the vessel endothelium merits dedicated investigation in future studies.

In conclusion, the Fe@POS nanoprobe, a gadolinium-free T1-weighted magnetic resonance contrast agent, possesses the potential for high-resolution and prolonged T1-weighted MRA of the cardiovascular system. Compared with Gd-DTPA, this novel Fe(III)-based nanoprobe has the following advantages: (1) extended vascular retention time, (2) high-spatial-resolution MRA, and (3) confirmed biosafety in animal experiments. This study improves the biocompatibility and *in vivo* circulation time of MR contrast agents and may provide a partial replacement for GBCAs in clinical practice.

## Supplementary information

**Additional file 1: Supplementary Fig. 1** Syntheses of Sar-NPC (A) and L-DOPA-NPC (B). **Supplementary Fig. 2** Diblock copolymerization of L-DOPA-NPC with Sar-NPC. **Supplementary Fig. 3** 1H NMR spectrum of Sar-NPC in DMSO-d6 (* H2O, ** DMSO). **Supplementary Fig. 4** 1H NMR spectrum of L-DOPA-NPC in DMSO-d6 (* DMSO, ** TMS, / diethyl ether). **Supplementary Fig. 5** 1H NMR spectrum of POS in deuterated dimethyl sulfoxide (DMSO-d6) (*H2O, **Dimethylacetamide, ***Tetramethyl silane, / Dimethyl sulfoxide). **Supplementary Fig. 6** Size exclusion chromatography traces (5 wt‰ in hexafluoroisopropanol) of POS and first block of POS(PDOPA10). **Supplementary Fig. 7** The nanoparticle size (a) and longitudinal relaxivity r1 (b) variation of Fe@POS nanoprobes in phosphate buffer solution (PBS) or Saline with endogenous cations (15 μmol/L Zn(II), 1 mmol/L Ca(II), 1 mmol/L Mg (II)) within 10 days (pH = 7.4, n = 3). The nanoparticle size (c) and longitudinal relaxivity r1 (d) variation of Fe@POS nanoprobes in serum extracted from BALB/c mice within 48 hours (37 ℃, n = 3). **Supplementary Fig. 8** Fluorescence images obtained by confocal laser scanning microscopy showing cellular uptake of Fe@POS nanoprobes in CT26 cells after incubation for 6 h. Scale bar: 10 μm. **Supplementary Fig. 9** ROIs used to evaluate the T1 signal intensity of rabbit aorta (red box) and kidney (blue circle) on a representative image. **Supplementary Fig. 10** CNR curve of the rabbit kidney over time. **Supplementary Fig. 11** ROIs used to evaluate the T1 signal intensity of thoracic aorta (red circle) and hepatic vein (blue box) on porcine models. **Supplementary Fig. 12** The short-term and acute toxicity evaluation of Fe@POS in BALB/c mice (n = 5) tested 24 h after intravenously treatment of Fe@POS (0.3 mmol Fe/kg). (a) white blood cells (WBC), (b) hematocrit (HCT), (c) hemoglobin (HGB), (d) lymphocyte count (LYM), (e) mean corpuscular hemoglobin (MCH), (f) mean corpuscular hemoglobin (MCHC), (g) mean corpuscular volume (MCV), (h) mean platelet volume (MPV), (i) platelet distribution width (PDW), (j) platelet - larger cell ratio (P-LCR), (k) red blood cells (RBC), (l) red blood cell distribution width (RDW-SD). (n = 5 biologically independent samples). **Supplementary Fig. 13** The short-term and acute toxicity evaluation of Fe@POS in Bama miniature pigs (n = 3) tested 24 h and 48 h after intravenously treatment of Fe@POS (0.06 Fe mmol/kg). (a) white blood cells (WBC), (b) hematocrit (HCT), (c) hemoglobin (HGB), (d) lymphocyte count (LYM), (e) mean corpuscular hemoglobin (MCH), (f) mean corpuscular hemoglobin (MCHC), (g) mean corpuscular volume (MCV), (h) mean platelet volume (MPV), (i) platelet distribution width (PDW), (j) platelet - larger cell ratio (P-LCR), (k) red blood cells (RBC), (l) red blood cell distribution width (RDW-SD). (n = 3 biologically independent samples).

## Data Availability

The main data of the article are provided as supplementary material in the form of Excel tables. The data in this article are accessible to anyone.

## Abbreviations

CNR: Contrast-to-noise ratio
CTA: Computed tomography angiography
FITC: Fluorescein isothiocyanate
GBCAs: Gadolinium-based contrast agents
Gd-DTPA: Gadolinium diethylenetriamine pentaacetic acid
MR: Magnetic resonance
MRA: Magnetic resonance angiography
POS: PolyDOPA-*b*-polysarcosine
ROI: Region of interest
SD: Standard deviation
SPIO: Superparamagnetic iron oxide
