# Supplementary material for: Magnetic resonance angiography based on iron-coordinated poly(amino acid)s nanoprobes: an in vitro and in vivo experimental study
Source: Eur Radiol Exp. 2026 Nov 6;10:172. doi: 10.1186/s41747-026-00829-5 (PMC13743709; doi:10.1186/s41747-026-00829-5)
Supplement: Supplementary file 1 [file 41747_2026_829_MOESM1_ESM.pdf]

Magnetic resonance angiography based on iron-coordinated poly (amino acids) nanoprobes: an *in vitro* and *in vivo* experimental study

ELECTRONIC SUPPLEMENTARY MATERIAL

Supplementary Material 1 Synthesis protocol of Fe@POS nanoprobe

One technician (S.X.) synthesized the monomers *N*-phenyloxycarbonyl-sarcosine (Sar-NPC) and *N*-phenyloxycarbonyl-3,4-dihydroxy-L-phenylalanine (L-DOPA-NPC). A suspension of sarcosine (26.7 g, 0.300 mol) and phenyl chloroformate (PCF, 37.3 mL, 0.300 mol) in 250 mL of ethyl acetate was heated at 45 ℃ for 48 hours. The reaction mixture was filtrated, evaporated under reduced pressure to get crude product which was purified by chromatography to obtain Sar-NPC as light-yellow viscous liquid (14.8 g, yield: 23.6%). L-DOPA-NPC was synthesized using the same method as Sar-NPC.

Diblock copolymerization of Sar-NPC and L-DOPA-NPC was performed using Schlenk technique. L-DOPA-NPC (2.40 g, 7.57 mmol) was dissolved in 12.0 mL of Dimethylacetamide (DMAc), followed by 0.757 g of neopentylamine (NPA) in DMAc solution. The vial was sealed and heated in a 60 ℃ bath for 24 hours. Then the polymerized L-DOPA block was transferred to a dimethylacetamide solution of Sar-NPC (6.63 g, 31.7 mmol) and DiPEA (0.537 g, 4.15 mmol). The mixture was heated in a 60 ℃ oil bath for 48 hours. The diblock copolymer was precipitated from diethyl ether and dried under vacuum ($PDOPA_x$-*b*-$PSar_y$, abbreviated as POS, 2.21 g, yield: 61.3%). The chemical structure of POS was characterized by ^1H Nuclear magnetic resonance spectra (^1H NMR) on a Bruker Avance DMX 400 spectrometer and size exclusion chromatography (SEC) instrument consisted of a Waters 1515 isocratic HPLC pump, a Waters 2414 interferometric refractometer and two Shodex KF series columns.

Fe@POS nanoprobe was prepared by a solvent exchange method using Schlenk technique. POS (308.0 mg) was dissolved in 5.0 mL DMSO. Then 5.0 mL DMSO solution of EtONa (20.2 mg, 0.300 mmol) and 5.0 mL DMSO solution of $Fe(NO_3)_3 \cdot 9H_2O$ (117.9 mg, 0.291 mmol) were added with continuous stirring. After 30 minutes, 30 mL deionized water was slowly added into the solution and the mixture was dialyzed against deionized water for 24 hours. Finally, the Fe@POS nanomicelles were obtained using ultrafiltration centrifugation technique. For the FITC labeled Fe@POS (FITC-Fe@POS), 5.0 mg FITC was added to the above reaction of POS and $Fe(NO_3)_3 \cdot 9H_2O$. The Fe@POS nanomicelle solution was lyophilized to obtain a powder, which was subsequently reconstituted into solutions at various concentrations for testing.

Supplementary Material 2 Cell Counting Kit-8 assay to evaluate the cytotoxicity of POS and Fe@POS

NIH 3T3 cells were seeded in a 96-well plate at the density of 1×10^4 cells per well and maintained in cell incubator for 24 h. Then, the medium was replaced with high-glucose DMEM medium containing POS and Fe@POS respectively with different concentrations (10, 50, 100, 200, 500, 1000 μg/mL). The cells were then incubated for another 24 hours, followed by the addition of 10 μL Cell Counting Kit-8 solution to each well. After 2 hours of incubation, the absorption value at 450 nm was measured by a microplate reader (BIO-RAD iMark) and the cell viability was quantified. The viability of cells treated without POS and Fe@POS was denoted as 100%. Three replicates were used for cytotoxicity assay.

Supplementary Material 3 Cellular uptake assay

Flow cytometry and confocal laser scanning microscopy were used to investigate the cellular uptake of Fe@POS. For the flow cytometry, CT26 cells were seeded in 12-well plates at the density of 2×10^5 cells per well and incubated for 24 hours. Then, the original medium was replaced with the high-glucose DMEM medium containing FITC-Fe@POS ([FITC] = 5 μg/mL), and the CT26 cells were further incubated for 2, 4 and 6 hours. After incubation, the CT26 cells were washed with phosphate buffer solution (PBS) for three times and detached from plates using 0.25% trypsin solution containing 0.02% EDTA. The CT26 cells were then collected into centrifugal tubes and analyzed by CytoFLEX LX flow cytometer and FlowJo V10 software. For the confocal laser scanning microscopy, CT26 cells (1×10^5 cells) were cultivated in glass-bottom dishes for 24 hours. Then, fresh high-glucose DMEM medium containing FITC-Fe@POS ([FITC] = 5 μg/mL) was added to replace the original medium, and CT26 cells were further incubated for 2, 4, and 6 hours. Subsequently, the cells were washed with PBS and stained with Hoechst 33342 and LysoTracker Red DND-99 for 30 minutes. Then, dye-labeled CT26 cells were observed by Nikon A1 confocal laser scanning microscope.

Supplementary Material 4 Relaxivity measurements

The relaxivity measurement experiments, the relaxivity of both Fe@POS and Gd-DTPA was measured in deionized water. For the relaxivity stability experiments, the relaxivity of Fe@POS was measured separately in PBS, saline with endogenous cations, and serum. Briefly, Fe@POS was dissolved in medium and diluted in a series of concentration gradients ([Fe] = 0.25, 0.20, 0.15, 0.10, 0.05 mM) before being encapsulated into 200 μL tubes. Inversion times were set as 50, 100, 200, 300, 400 and 600 milliseconds for all samples. The Longitudinal relaxivity r1 was obtained by linearly fitting the reciprocal of T1-time to concentration. Gd-DTPA was applied as a control.

Supplementary Material 5 MRI of New Zealand rabbits

Rabbits were anesthetized via intramuscular injection of Zoletil (10 mg/kg, Tiletamine Hydrochloride and Zolazepam Hydrochloride, Virbac, France) and xylazine Hydrochloride (2 mg/kg, Shengda Animal). To evaluate the MRI enhancement of Fe@POS nanoprobe, rabbits were intravenously injected with Fe@POS or Gd-DTPA. The MR images were acquired before and after administration at different time points. The MR signal of aorta in New Zealand rabbits was measured. Contrast-to-noise ratio (CNR) values were calculated as follows:

$$CNR = (SI_{ROI} - SI_{tissue})/SD_{background}$$

where SI_{ROI} is the signal intensity of region of interest (ROI), SI_{tissue} is the signal intensity of tissues adjacent to aorta, $SD_{background}$ is the standard deviation (SD) of the background signal from non-tissue regions.

The aorta signal retention values were calculated as follows:

$$Retention_{aorta} = (SI_{ROI} - SI_{pre})/(SI_{max} - SI_{pre}) \times 100\%$$

where SI_{ROI} is the signal intensity of region of interest, SI_{pre} is the signal intensity of aorta before Fe@POS administration, SI_{max} represents the maximum signal intensity of aorta.

We used a generic MR Liver Acquisition with Volume Acceleration–LAVA sequence to acquire MR signals from the thoracoabdominal aorta and kidneys in rabbits. The parameters of the sequence are as follows: repetition time ms/echo time ms) = 3.8/1.8, slice thickness = 1.0 mm, flip angle = 3 degree, field of view = 80 × 80 mm^2, matrix size = 256 × 256.

Supplementary Material 6 MRA of Bama swine

Bama swine models were anesthetized via intramuscular injection of Zoletil (10 mg/kg) and Atropine Sulfate (0.05 mg/kg, Huamu Animal). Anesthesia was maintained with Propofol (5 mg/kg per hour). Then, Bama pigs were intravenously administrated with Fe@POS nanoprobes (0.06 mmol Fe/kg). The MR signals of thoracic aorta and hepatic vein were acquired before and after Fe@POS administration at 0, 1, 2, 3 hours. CNR values of thoracic aorta and hepatic vein were calculated as follows:

$$CNR = (SI_{ROI} - SI_{tissue})/SD_{background}$$

where SI_{ROI} is the signal intensity of ROI in thoracic aorta or hepatic vein, SI_{tissue} is the signal intensity of tissues adjacent to aorta/vein, $SD_{background}$ is the standard deviation of the background signal from non-tissue regions.

The parameters of MRA protocol are as follows: repetition time ms/echo time ms) = 3.45/1.32, slice thickness = 3.0 mm, field of view = 380 × 380 mm^2. The MR images were processed by a RadiAnt DICOM viewer.

Supplementary Material 7 Biodistribution analysis

Female BALB/c mice (22.4 ± 1.3 g body weight, mean ± standard deviation) were randomly divided into five experimental groups (n = 3 for each experimental group) and one control group (n = 3). The mice of experimental group were intravenously administrated with Fe@POS (0.1 mmol Fe/kg), whereas the mice of control group were intravenously injected with the equal volume of saline. Then, the mice of experimental groups were killed by cervical dislocation under deep isoflurane anesthesia at 12, 24, 48, 96 and 168 hours time points, subsequently followed by the dissection and collection of major organs (heart, lung, liver, spleen and kidney). Organ samples were weighed and predigested in 6.0 mL concentrated nitric acid for 48 h and digested at 250 ℃ for 1 hour. The remaining clear solution was diluted to 6.0 mL with 1% HNO3 and the Fe concentration was quantified by inductively coupled plasma-mass spectrometry (ICP-MS).

Supplementary Material 8 Serum biochemical and hematological analysis

For the serum biochemical and hematological analysis, healthy female BALB/c mice (21.4 ± 0.9 g body weight) were randomly divided into two experimental groups (n = 4 for each experimental group) and one control group (n = 4). The mice of two experimental groups were intravenously injected with Fe@POS (0.3 mmol Fe/kg) every three days. The mice of control group were intravenously injected with saline every three days. At day 7 and 14, mice were killed by cervical dislocation under deep isoflurane anesthesia. Peripheral blood and serum samples were collected for subsequent biochemical and hematological tests. Additionally, we investigated the short-term and acute toxicity of Fe@POS nanoprobes in rodent and porcine models. A total of 10 healthy female BALB/c mice (20.8 ± 0.8 g body weight) were randomly divided into one experimental group (n = 5) and one control group (n = 5). The mice of experimental group were intravenously injected with Fe@POS (0.3 mmol Fe/kg) and the mice of control group were

intravenously injected with saline. All mice were killed after 24 hours and blood samples were collected for hematological analysis. In the porcine group (n = 3, 30.3 ± 2.1 kg body weight), 2 mL blood was collected from the marginal ear vein before Fe@POS injection, and at 24 hours and 48 hours after the injection of the Fe@POS nanoprobes (0.06 mmol Fe/kg). All biochemical and hematological tests were conducted by a bio-testing company (Haoke Biotechnology Co. Ltd).

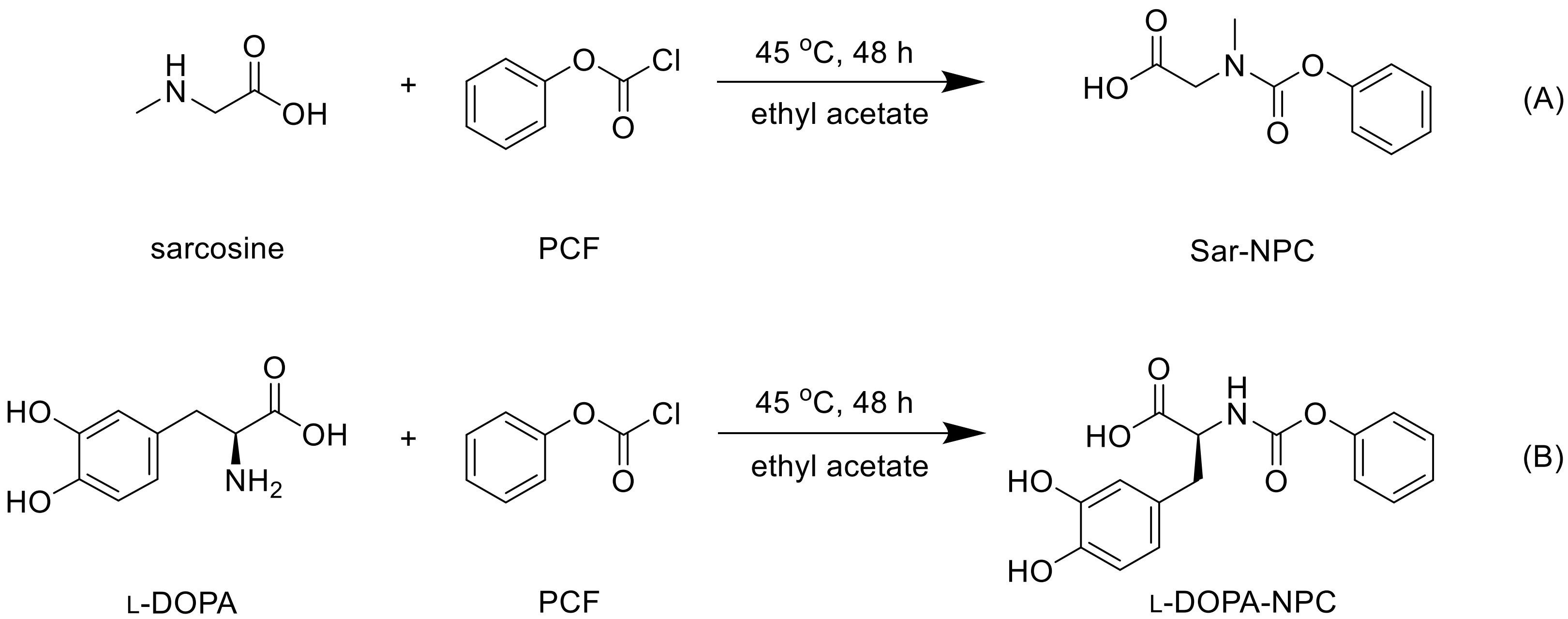

Supplementary Fig. 1 Syntheses of Sar-NPC (A) and L-DOPA-NPC (B).

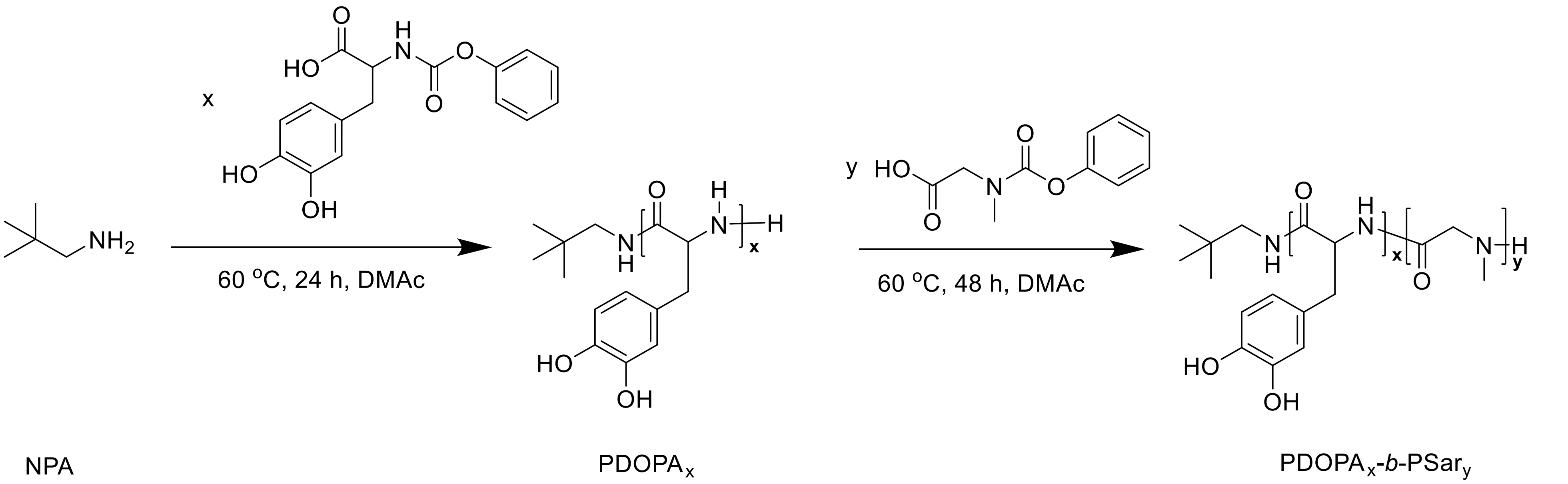

Supplementary Fig. 2 Diblock copolymerization of L-DOPA-NPC with Sar-NPC.

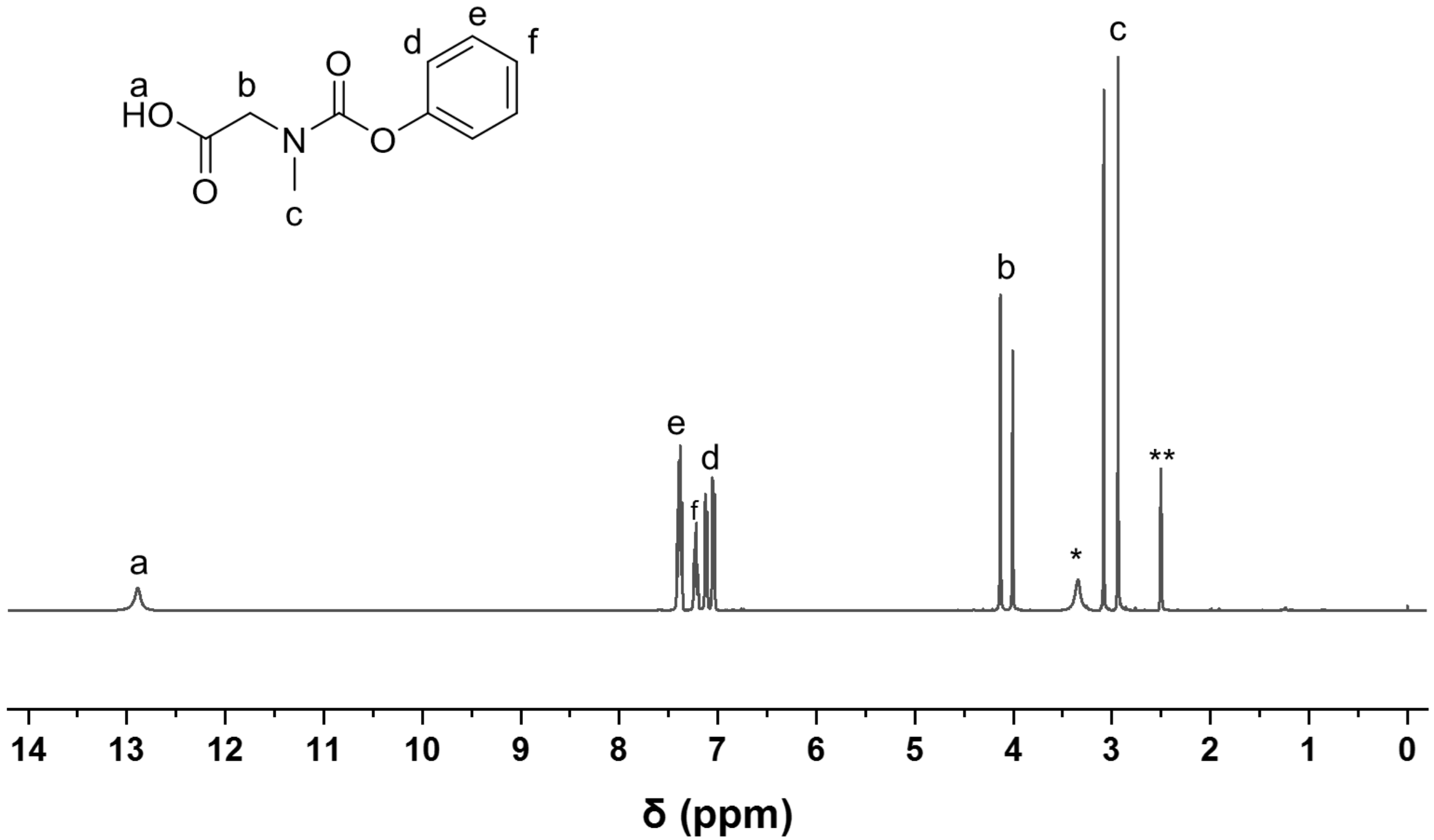

Supplementary Fig. 3 ^{1}H NMR spectrum of Sar-NPC in DMSO-d_6 (* H_2O, ** DMSO).

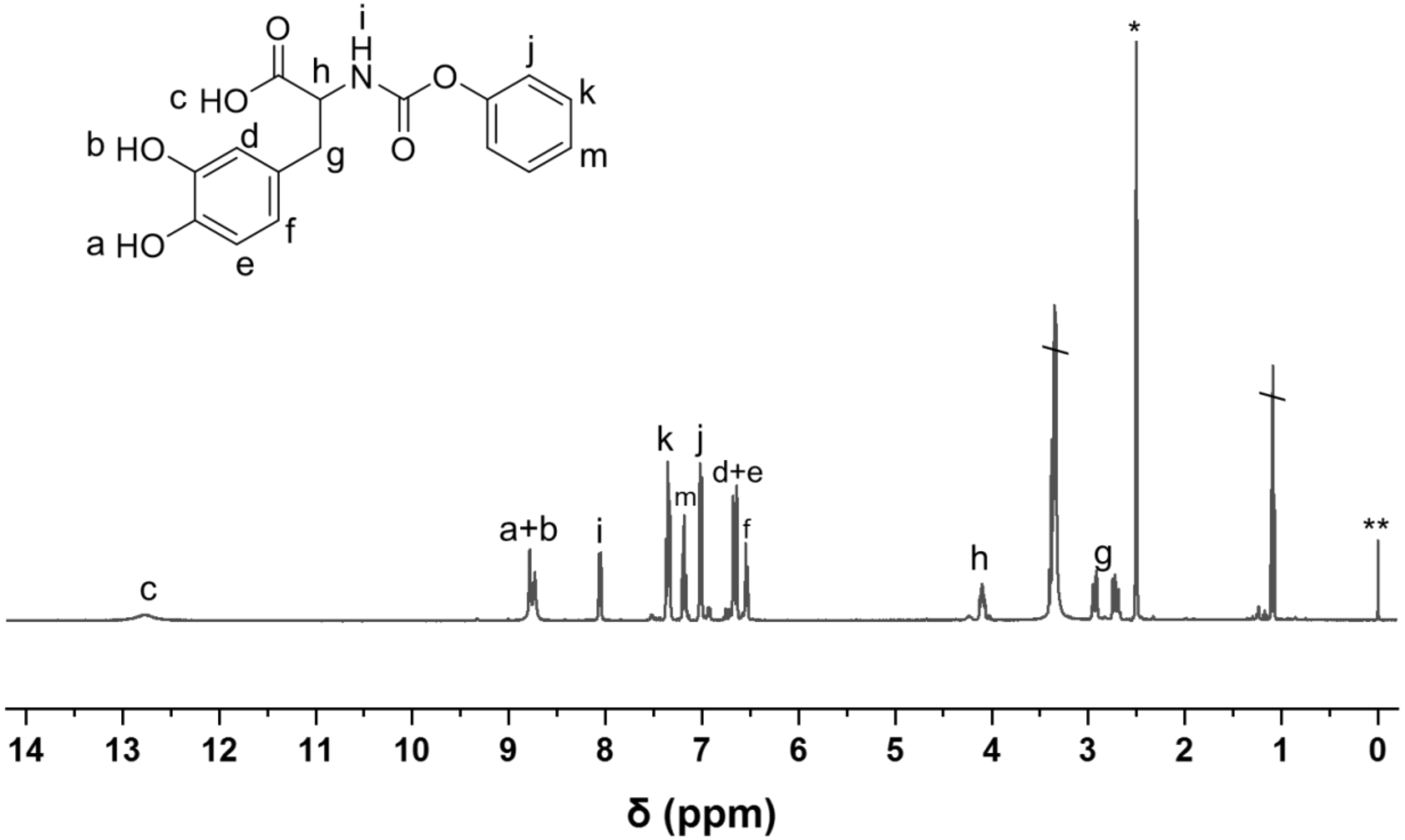

Supplementary Fig. 4 ^{1}H NMR spectrum of L-DOPA-NPC in DMSO-d_6 (* DMSO, ** TMS, / diethyl ether).

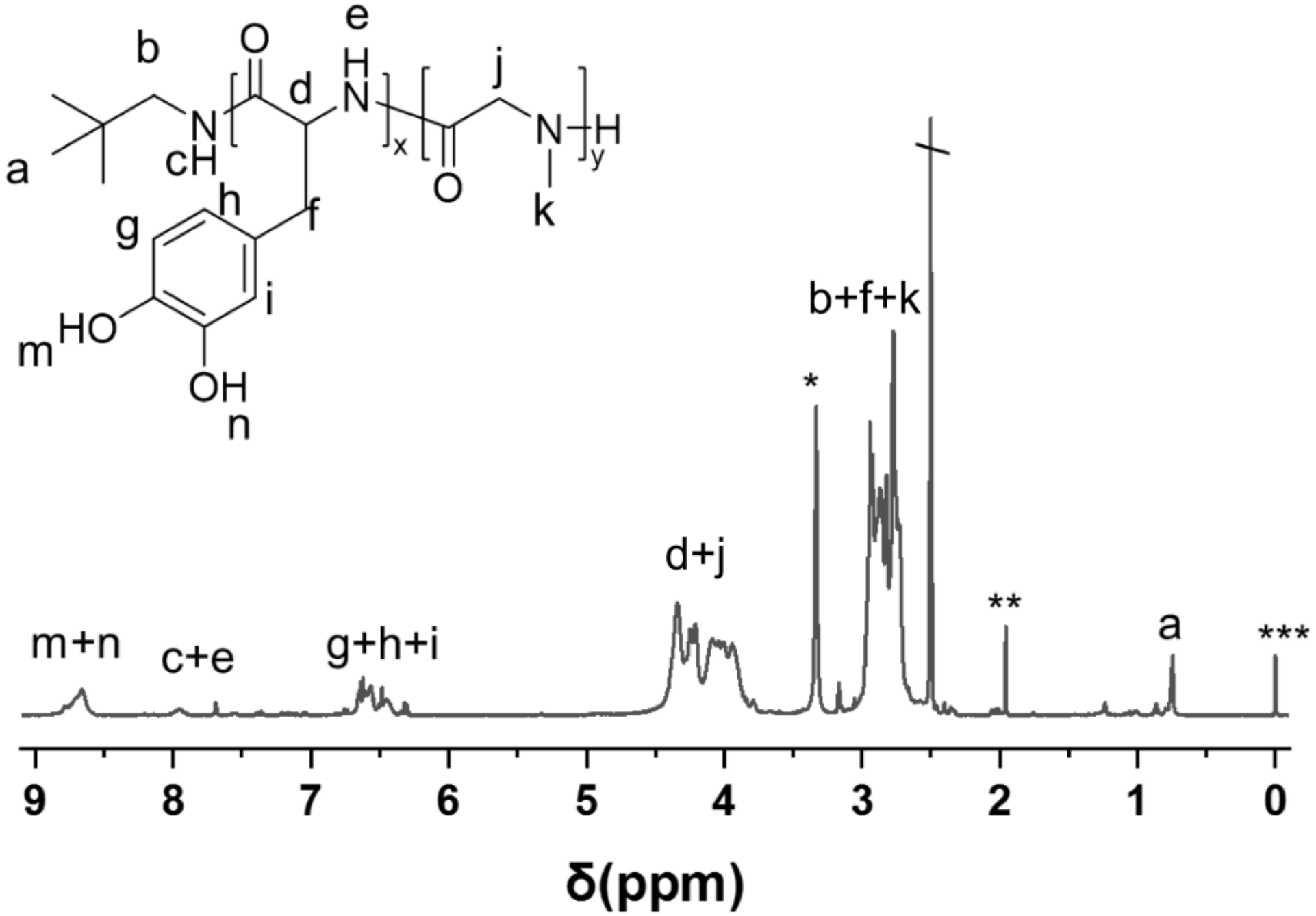

Supplementary Fig. 5 ^{1}H NMR spectrum of POS in deuterated dimethyl sulfoxide (DMSO-d_6) (*H_2O, **Dimethylacetamide, ***Tetramethyl silane, / Dimethyl sulfoxide).

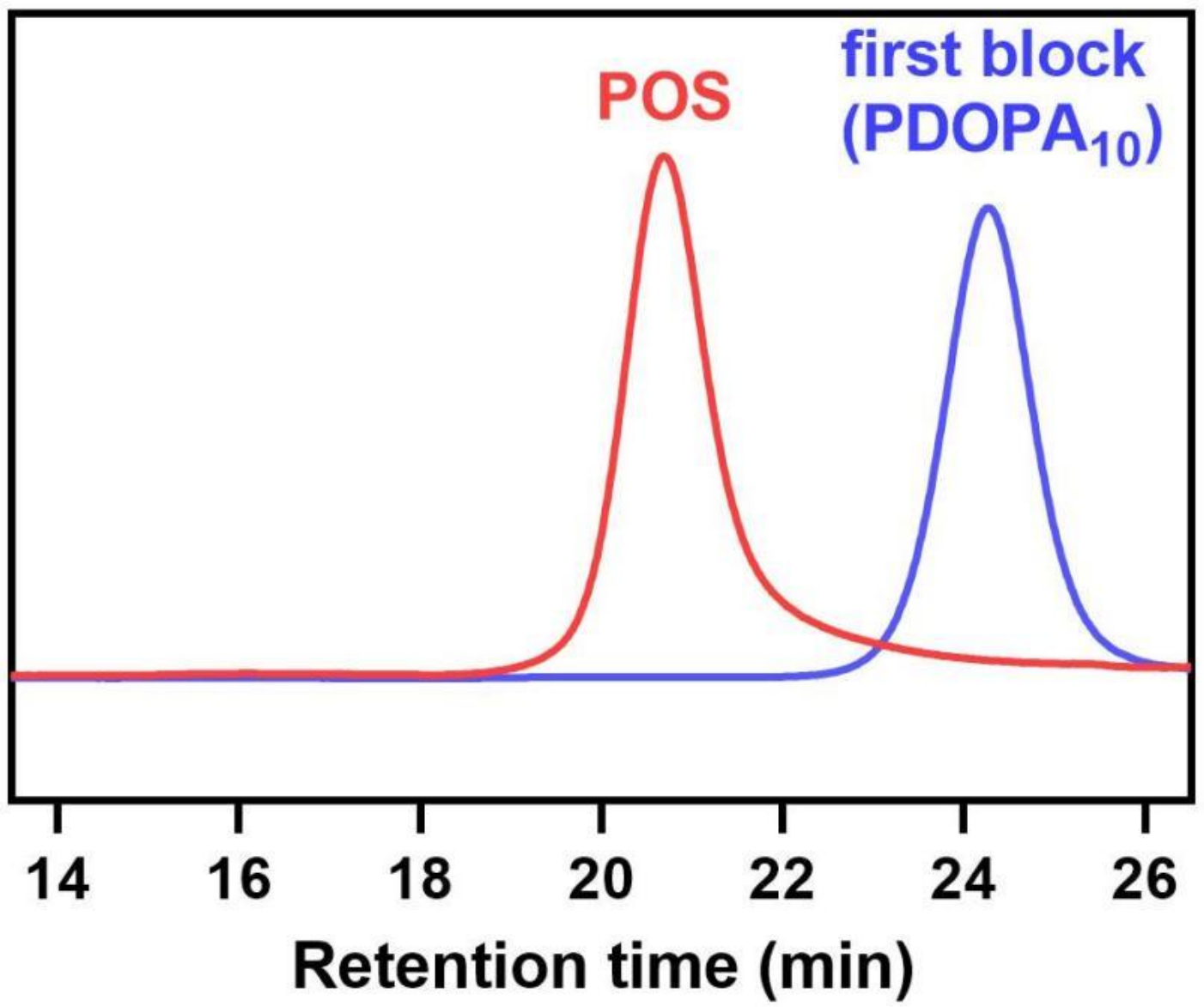

Supplementary Fig. 6 Size exclusion chromatography traces (5 wt‰ in hexafluoroisopropanol) of POS and first block of POS($PDOPA_{10}$).

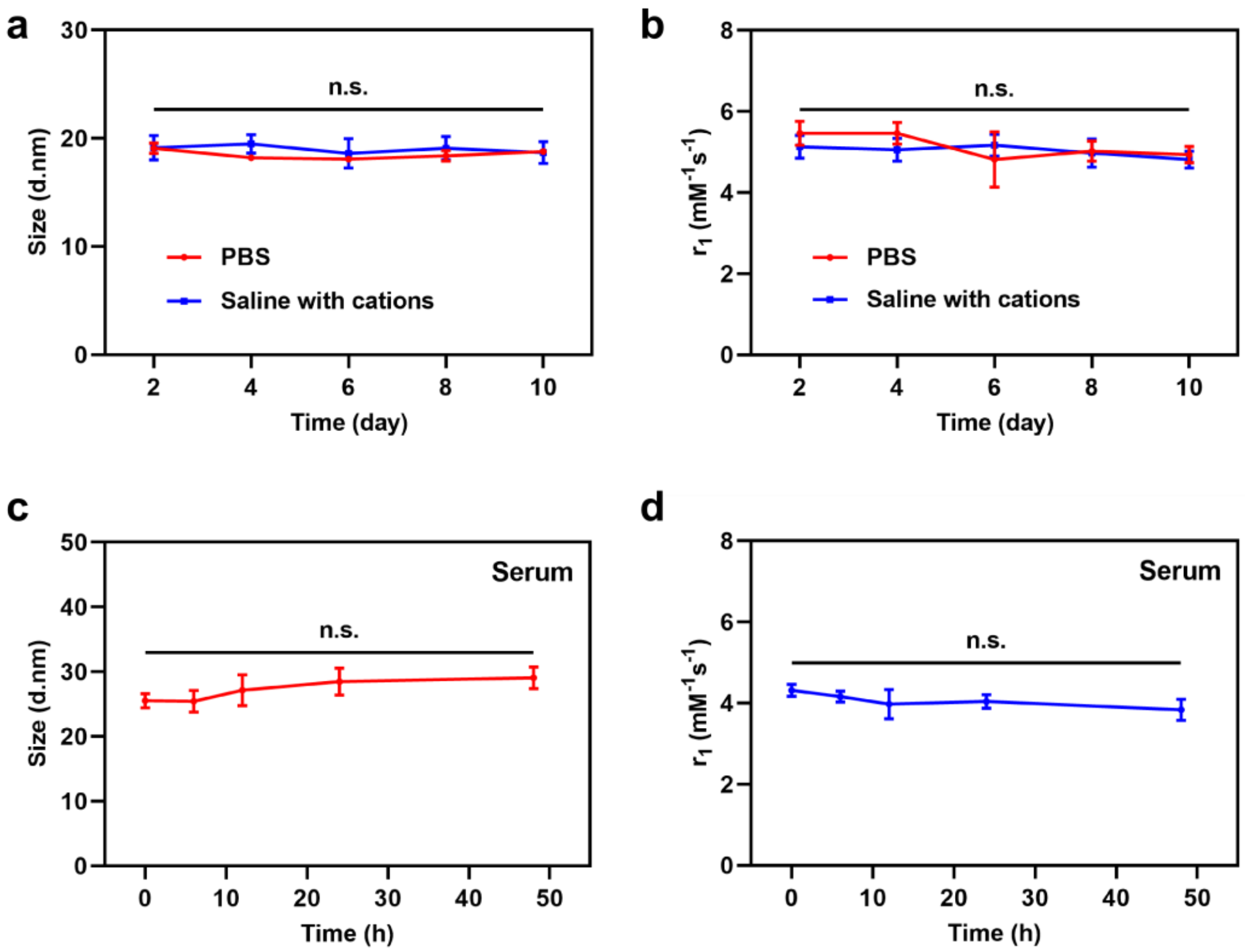

Supplementary Fig. 7 The nanoparticle size (a) and longitudinal relaxivity r_1 (b) variation of Fe@POS nanoprobes in phosphate buffer solution (PBS) or Saline with endogenous cations (15 μmol/L Zn(II), 1 mmol/L Ca(II), 1 mmol/L Mg (II)) within 10 days (pH = 7.4, n = 3). The nanoparticle size (c) and longitudinal relaxivity r_1 (d) variation of Fe@POS nanoprobes in serum extracted from BALB/c mice within 48 hours (37 ℃, n = 3).

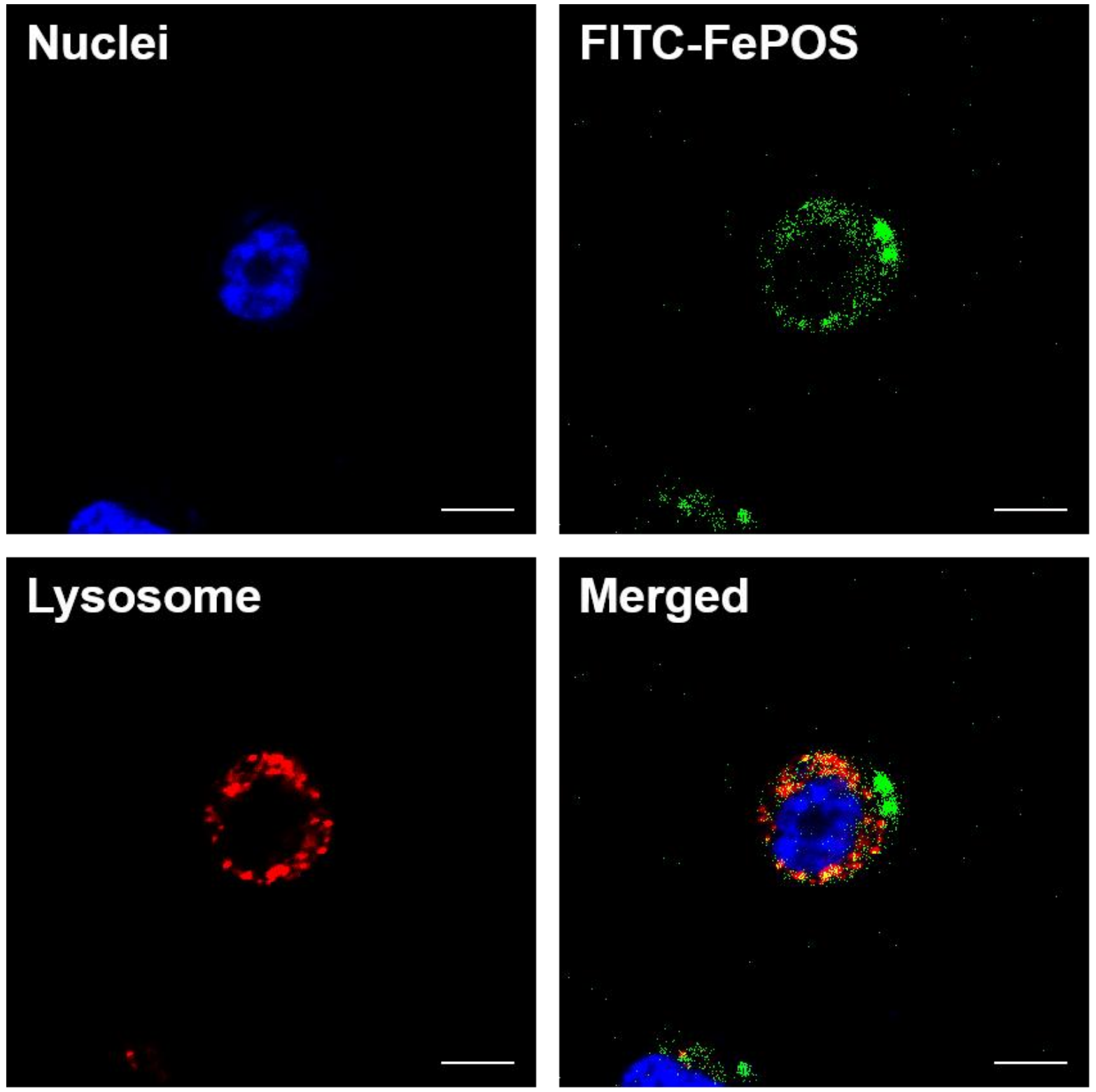

Supplementary Fig. 8 Fluorescence images obtained by confocal laser scanning microscopy showing cellular uptake of Fe@POS nanoprobes in CT26 cells after incubation for 6 h. Scale bar: 10 μm.

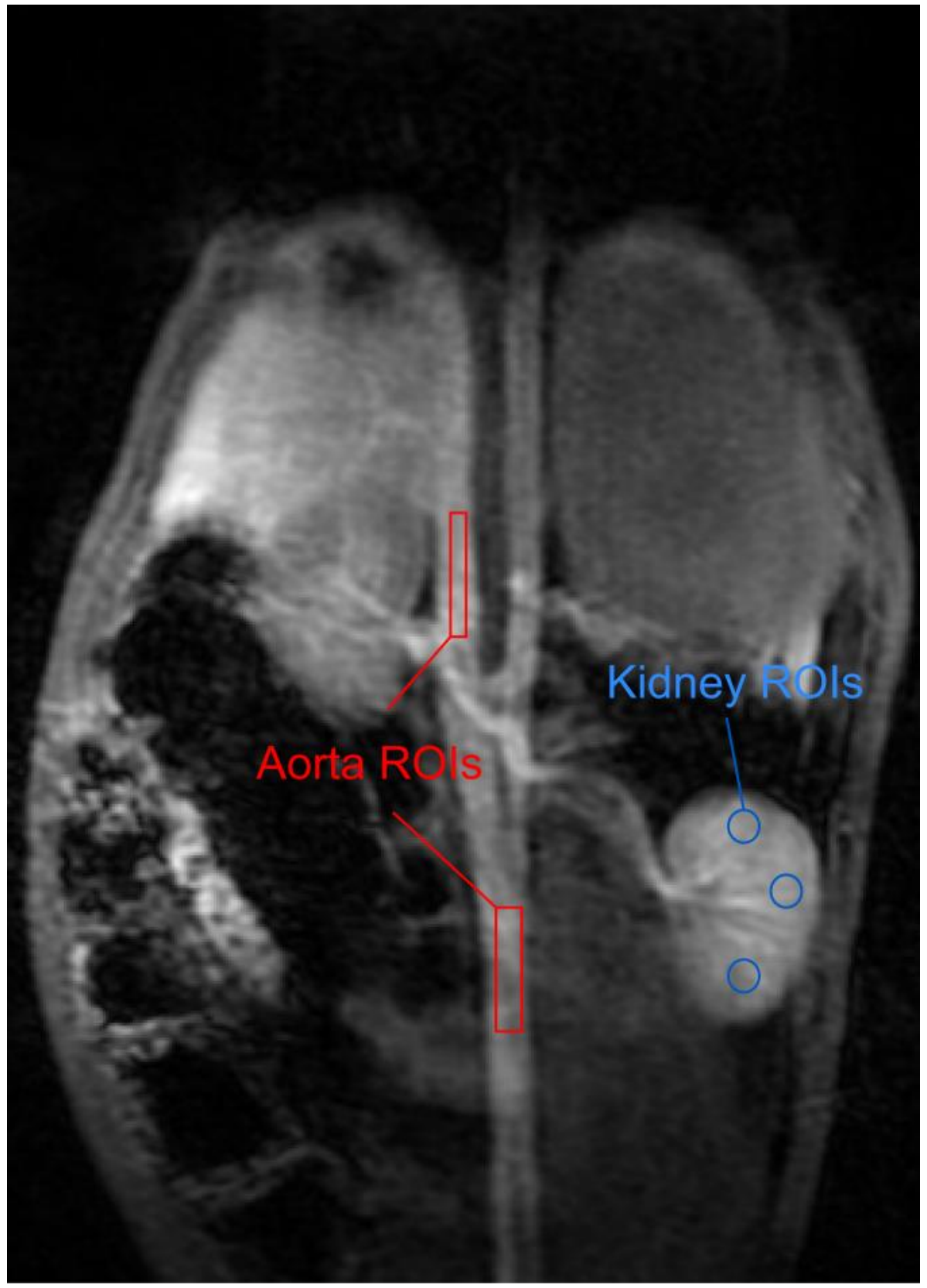

Supplementary Fig. 9 ROIs used to evaluate the T1 signal intensity of rabbit aorta (red box) and kidney (blue circle) on a representative image.

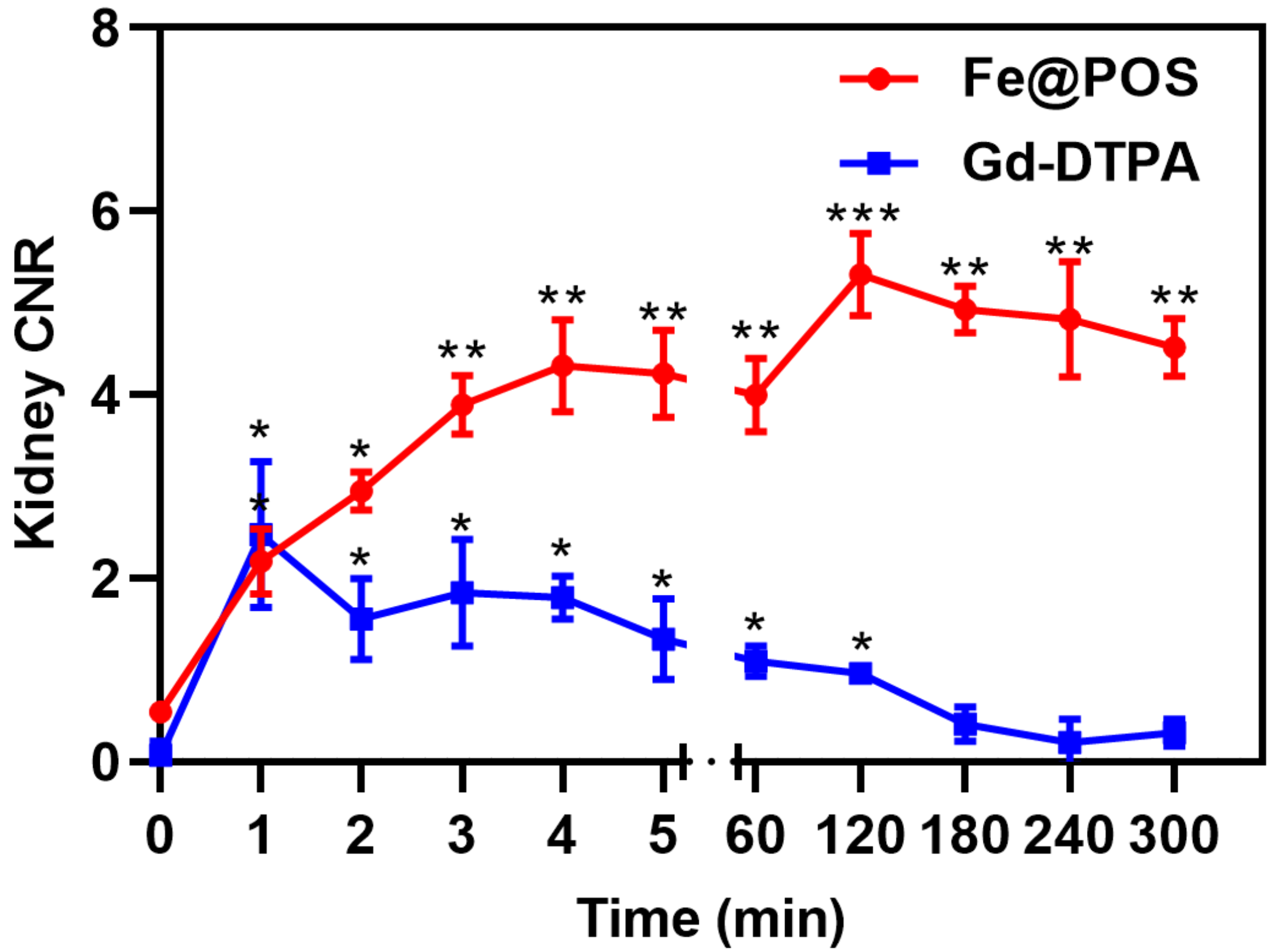

Supplementary Fig. 10 CNR curve of the rabbit kidney over time.

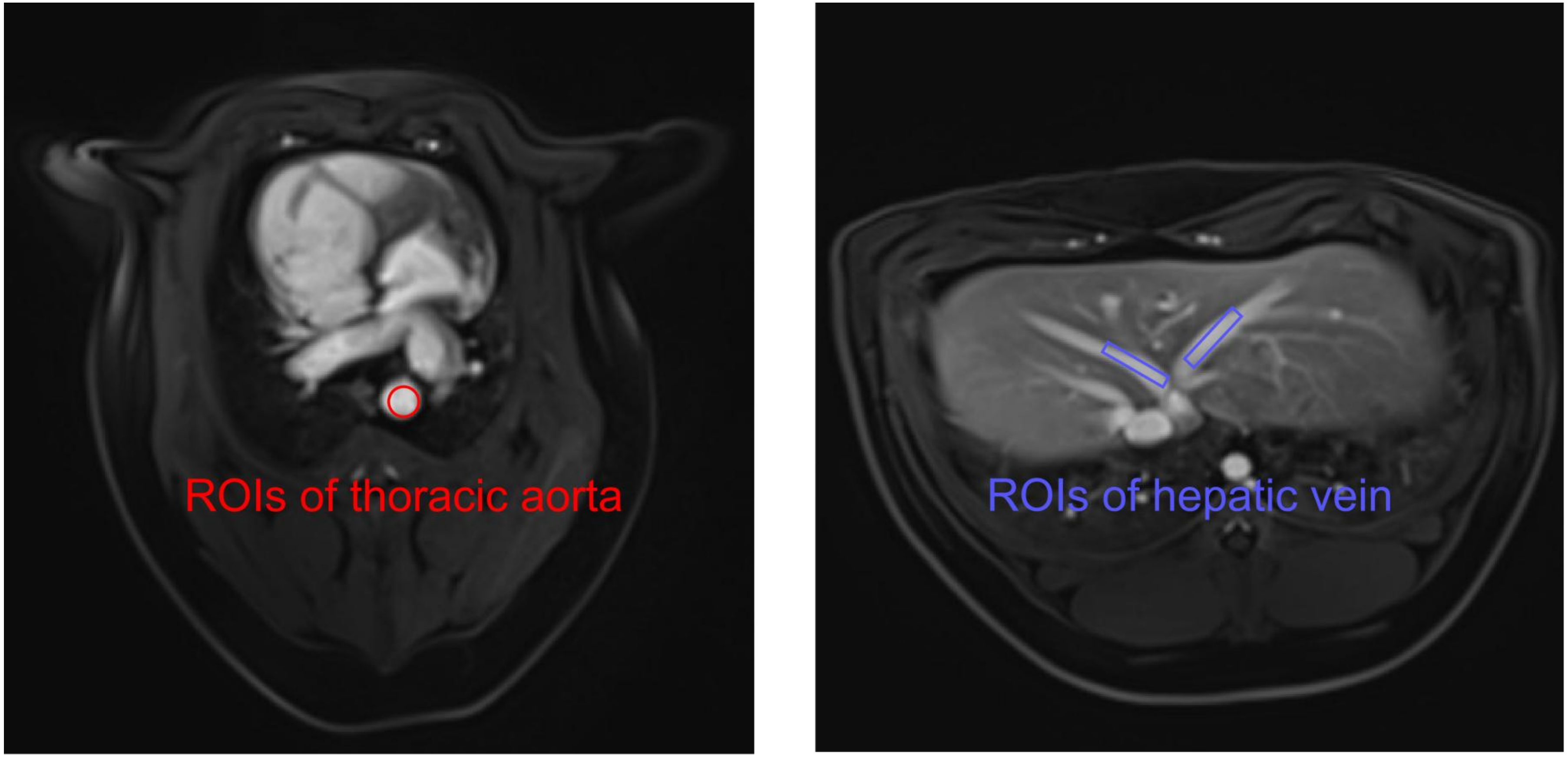

Supplementary Fig. 11 ROIs used to evaluate the T1 signal intensity of thoracic aorta (red circle) and hepatic vein (blue box) on porcine models.

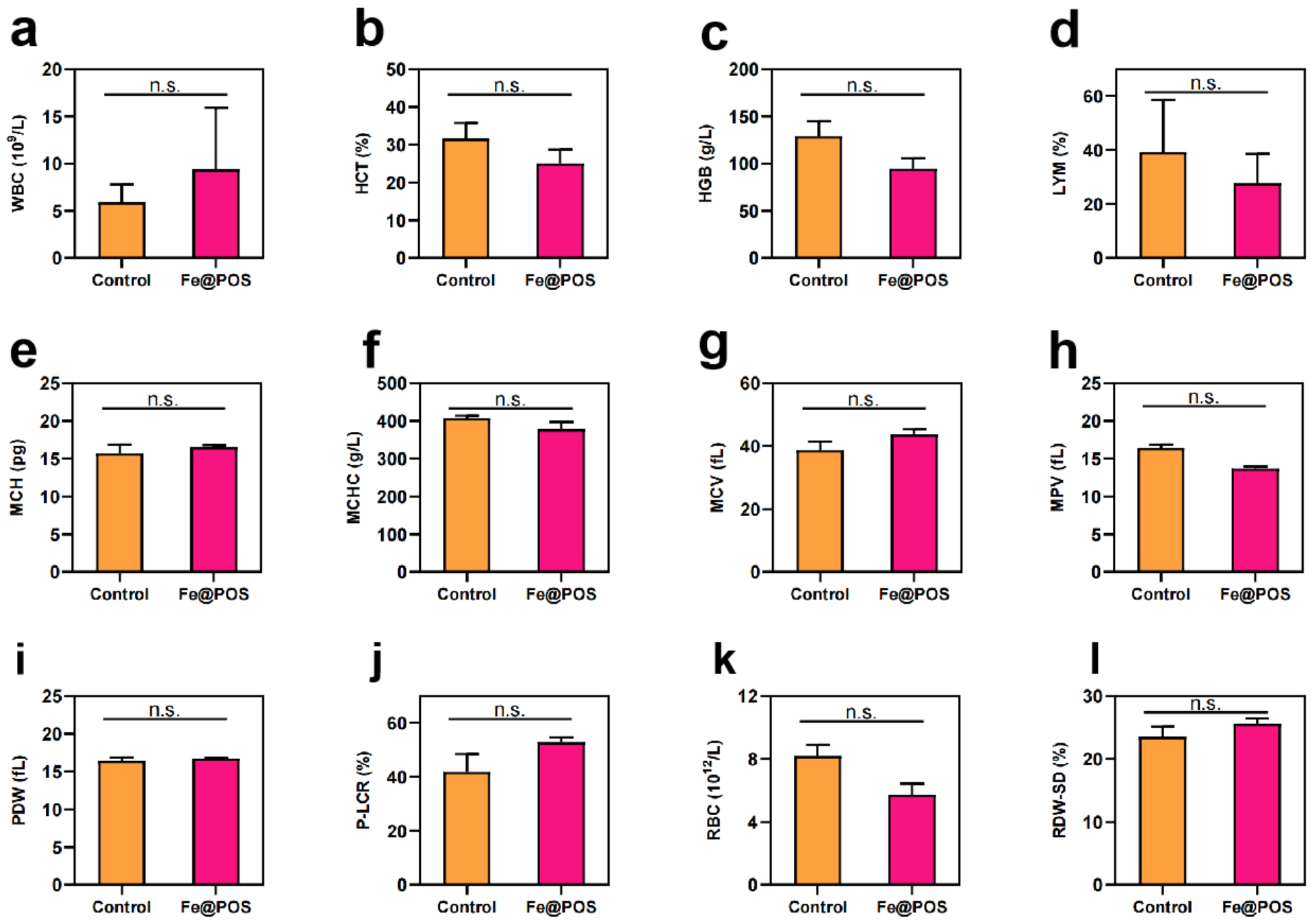

Supplementary Fig. 12 The short-term and acute toxicity evaluation of Fe@POS in BALB/c mice (n = 5) tested 24 h after intravenously treatment of Fe@POS (0.3 mmol Fe/kg). (a) white blood cells (WBC), (b) hematocrit (HCT), (c) hemoglobin (HGB), (d) lymphocyte count (LYM), (e) mean corpuscular hemoglobin (MCH), (f) mean corpuscular hemoglobin (MCHC), (g) mean corpuscular volume (MCV), (h) mean platelet volume (MPV), (i) platelet distribution width (PDW), (j) platelet - larger cell ratio (P-LCR), (k) red blood cells (RBC), (l) red blood cell distribution width (RDW-SD). (n = 5 biologically independent samples).

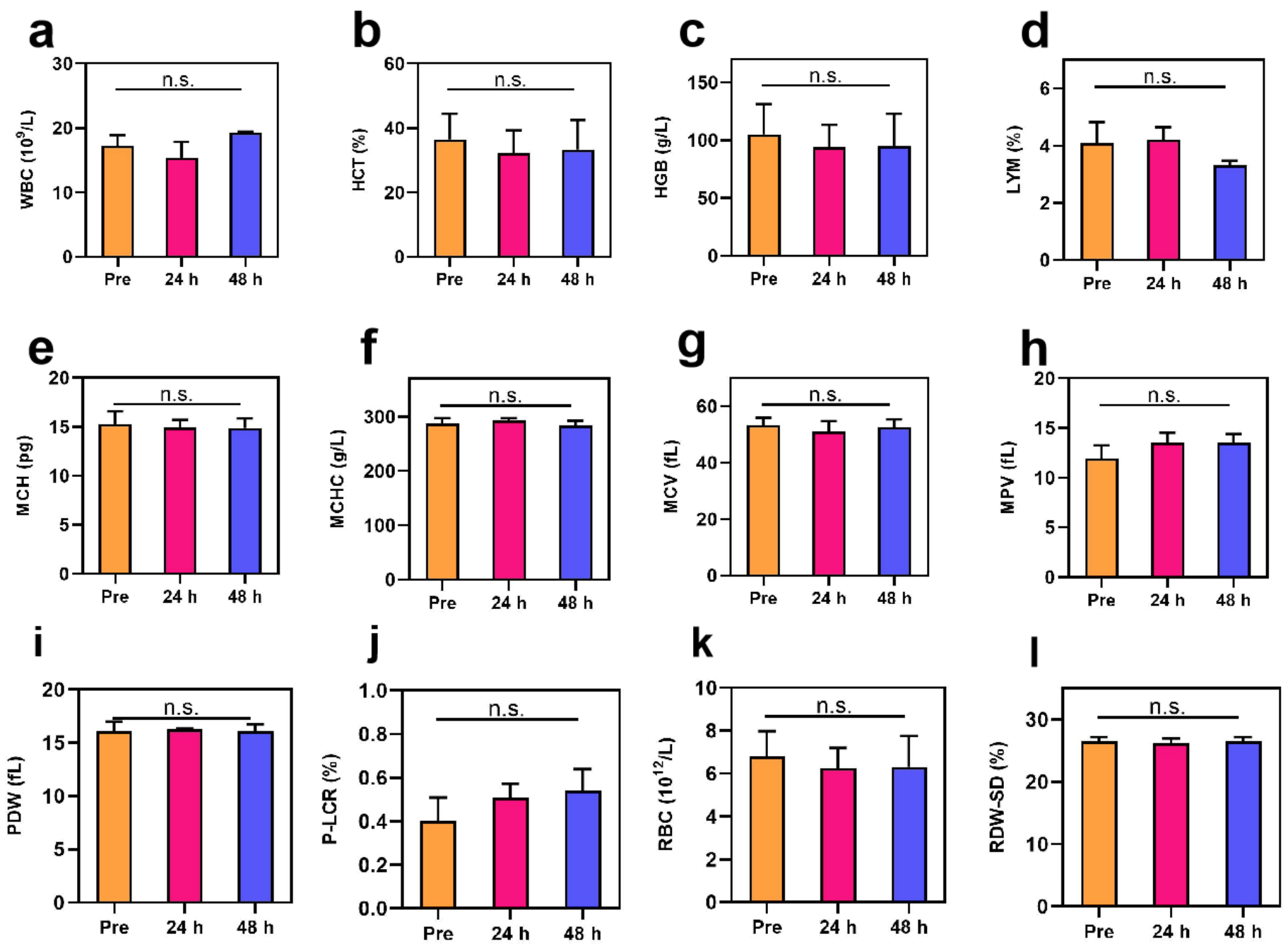

Supplementary Fig. 13 The short-term and acute toxicity evaluation of Fe@POS in Bama miniature pigs (n = 3) tested 24 h and 48 h after intravenously treatment of Fe@POS (0.06 Fe mmol/kg). (a) white blood cells (WBC), (b) hematocrit (HCT), (c) hemoglobin (HGB), (d) lymphocyte count (LYM), (e) mean corpuscular hemoglobin (MCH), (f) mean corpuscular hemoglobin (MCHC), (g) mean corpuscular volume (MCV), (h) mean platelet volume (MPV), (i) platelet distribution width (PDW), (j) platelet - larger cell ratio (P-LCR), (k) red blood cells (RBC), (l) red blood cell distribution width (RDW-SD). (n = 3 biologically independent samples).

Supplementary original data

Release profile (Figure 1d)

time(h)	Fe release (%)		
0,5	0,5377	0,5851	0,5264
1	0,9278	1,1573	1,0924
2	1,4425	1,8046	1,6453
4	1,7103	2,4333	2,0813
8	2,0341	3,0112	3,045
12	2,1739	3,3274	3,47
24	2,2613	3,7462	4,1352
48	2,4712	4,0269	4,5742

R1 value (figure 1f)

	1/T1 (s-1)	
Fe/Gd concentration (mM-1s-1)	Fe@POS	Gd-DTPA
0,05	1,0359152	0,9124088
0,1	1,2922735	1,080497
0,15	1,5603302	1,2518778
0,2	1,8840197	1,5459535
0,25	2,1310602	1,7752215

Cell viability (figure 2a)

Fe@POS			
con.(μg/mL)			
10	110	97,30263158	103,1578947
50	101,0526316	103,2236842	101,5131579
100	110,5921053	102,8289474	100,7894737
200	99,80263158	97,89473684	96,84210526
500	97,82894737	97,10526316	86,05263158
1000	99,73684211	100,7894737	97,96052632

POS			
con.(μg/mL)			
10	109,1616249	101,9014693	113,9152982
50	117,7182368	103,5436474	108,1244598
100	92,13483146	119,7061366	113,9152982
200	101,642178	113,0509939	104,7536733
500	91,01123596	99,22212619	101,8150389
1000	99,74070873	113,4831461	107,8651685

MRI analysis (Figure 3 and 4)

Gd-DTPA	CNR			SNR			signal retention rate(%)		
0	0,498098859	0,513851168	0,47148289	4,839761	3,827810972	3,87017925	4,759109233	-2,532190521	-2,226918712
1	13,34111896	11,44703965	14,73221076	17,682781	15,78870179	19,0738729	97,29560487	83,6483895	107,3186959
2	8,102118414	6,546985334	5,940793047	12,443781	10,88864747	10,28245519	59,54757152	48,34253063	43,97479551
3	6,136338946	6,631178707	4,860945139	10,478001	10,97284085	9,202607279	45,38374232	48,9491605	36,19427811
4	4,013036393	1,145573058	2,950570342	8,3546985	5,487235198	7,292232482	30,08492818	9,424288678	22,4296505
5	1,370450842	2,377512222	2,346550788	5,712113	6,719174362	6,688212928	11,04457751	18,30065359	18,07757035

6	1,62629006	1,925583922	0,965236285	5,9679522	6,267246062	5,306898425	12,88794959	15,04442096	8,124926617
30	1,073872895	0,48940793	0,504617056	5,415535	3,85225421	4,846279196	8,907674846	-2,356072169	4,806074126
60	0,69744704	0,009777295	0,407930473	5,0391092	4,331884845	4,749592613	6,195452233	1,099761262	4,109428202
120	0,831613254	0,431830527	0,78109723	5,1732754	3,909831613	5,12275937	7,162146296	-1,941215608	6,798168369
180	0,964149919	0,260184682	0,349266703	5,3058121	4,081477458	3,992395437	8,117099135	-0,704473406	-1,346326954
240	0,600760456	0,004345464	0,422596415	4,9424226	4,337316676	3,919065725	5,498806309	1,138898673	-1,874682009
300	0,712112982	0,034763715	0,464964693	5,0537751	4,306898425	3,876697447	6,301123244	0,919729169	-2,179953818

Bama swine		

time (min)	CNR		
pre	0,33333333	0,75555555	0,44444444
1	40,04444444	47,31111111	46,31111111
3	36,95555556	32,95555556	36,77777778
5	32,73333333	33,06666667	25,04444444
60	32,26666667	28,86666667	31,95555556
120	25,95555556	28,86666667	31
180	31	29,55555556	25,6

Bama swine			
time (min)	CNR		
pre	10,67777777	8,8	7,28888888
1	9,86666666	7,37777777	6,11111111
3	41,6	55,04444444	43,48888889
5	39,48888889	50,57777778	53,57777778
60	15,6	33,11111111	21,71111111
120	16,11111111	16,68888889	21,57777778

150	12,88888889	11,88888889	14,2

Biosafety evaluation (Figure 5)

body weight change							
healthy							
day1	day3	day5	day7	day9	day11	day13	day15
22,04	23,32	23,78	23,96	22,88	24,83	25,07	25,16
21,28	22,11	21,58	23,42	23,28	22,41	21,59	23,75
21,15	21,45	23,32	23,38	21,44	24,46	22,90	24,13

21,15	21,20	22,41	21,66	21,19	20,43	24,02	24,53

0.1mmol/kg							
day1	day3	day5	day7	day9	day11	day13	day15
22,61	22,88	22,58	23,45	23,71	24,71	24,41	25,25
22,36	21,66	20,16	23,75	22,94	22,40	22,54	22,29
20,81	21,33	23,81	22,61	20,18	24,93	24,65	22,87
19,12	21,05	21,05	20,59	21,12	21,66	21,95	21,99

0.3mmol/kg							
day1	day3	day5	day7	day9	day11	day13	day15
20,75	19,75	19,68	20,72	21,27	22,56	22,93	23,04
21,69	22,33	22,31	23,07	22,98	22,20	22,49	22,21
21,91	22,53	20,43	22,30	21,14	21,82	21,72	22,67
21,85	23,79	23,24	23,54	23,80	24,40	24,87	24,35

Serum biochemical analysis of hepatotoxicity												
	Day 0				Day 7				Day 14			

ALP	6	10,5	7,3	18,7	11,1	9,6	10,5	15,9	11,1	9,6	10,5	15,9
ALT	33,8	51,1	44,6	43,8	82,7	54,3	35,4	60,2	43,4	73,8	35	75,9
AST	117,9	128,5	161,5	98,7	117,2	120,2	109,7	135	144,2	170,5	158,5	111,3
	Day 0				Day 7				Day 14			
CR	33,8	34,2	34	35,4	32,8	33,7	32,9	32,6	33,2	35,2	33	35,6
UA	21	44,3	38,7	51,8	59,6	72,5	61,4	41,9	75,2	80,9	96,8	43,7
BUN	4953	7786	6755	6649	6228	5605	5610	7689	7561	7653	5082	6530
	Day 0				Day 7				Day 14			

ALB	27,9	31,2	33	32,5	25,8	28,5	32,9	30,6	26,1	32,2	29,6	28,8
GLB	30,3	31	20,3	28,6	33,9	31,5	25,7	28,1	21,6	23,1	23,8	21,2
TP	58,2	62,2	53,3	61,1	59,7	60	58,6	58,7	47,7	55,3	53,4	50

Hematological analysis of peripheral blood												
	Day 0				Day 7				Day 14			
MPV	15,8	15	11	12,2	12,1	12,3	13,2	11,5	14,6	11,8	15,7	11,6
RDW-SD	25,6	26	24,6	25,8	23,1	24,4	26,5	25,4	26,4	25,1	27,4	24,2
MCV	37,3	48	45,8	43,6	34,4	47,1	38,8	38,3	38,7	35,6	34,2	42,6

	Day 0				Day 7				Day 14			
WBC	3,99	6,52	3,91	3,73	6,41	7,96	6,65	4,95	5,03	4,51	6,6	6,94
RBC	7670	8460	7370	8400	7850	6410	6190	8430	8390	5530	6520	6620
	Day 0				Day 7				Day 14			
HGB	149	147	160	143	112	150	164	126	169	131	127	165
MCHC	327	334	373	377	331	396	394	338	443	398	345	438
	Day 0				Day 7				Day 14			
LYM	1,19	0,89	3,49	1,66	2,57	4,4	3,98	3,81	2,02	3,1	1,94	3,21

PDW	15,9	15,5	16,9	16,3	16,1	16	16,5	16,7	16,8	16,4	16,6	16,2
HCT	29,6	28	31,7	28,1	34,3	31	31,3	25	31,9	27	25,9	26,4
